# Supplementary material for: Effect of Orthopedic Treatment for Class III Malocclusion on Upper Airways: A Systematic Review and Meta-Analysis
Source: J Clin Med. 2020 Sep 18;9(9):3015. doi: 10.3390/jcm9093015 (PMC7563370; doi:10.3390/jcm9093015)
Supplement: Supplementary file 1 [file jcm-09-03015-s001.pdf]

## Supplementary Materials

**Supplementary Material S1.** Additional review details and deviations from the protocol.

| |
|---|
| **Deviations from the protocol** |
| The SMD was decided post hoc to be used post-hoc to pool 4 studies with slightly different cephalometric measurements of total nasopharyngeal area. |
| Several factors were planned to be assessed through subgroup analyses/meta-regressions in meta-analyses of at least 5 studies, but they could not be ultimately conducted due to limited material/reporting: (i) subsets according to the patient characteristics (patient skeletal age, ethnicity, craniofacial configuration, masticatory activity, etc.), (ii) subsets according to variations of administered treatment and any co-interventions like use of extraoral appliances, skeletal anchorage, full fixed appliances, etc, (iii) subsets according to patient compliance with the treatment. |
| Robustness of the results was planned a priori to be checked a priori with sensitivity analyses based on (a) inclusion/exclusion of non-randomized studies, (b) inclusion/exclusion of trials with methodological shortcomings, and (c) improvement of the GRADE classification. No such sensitivity analyses could be performed. |
| **Additional methods** |
| Forest plots were augmented with contours denoting effect size for orthopedic treatment, according to the average Standard Deviation (SD) pre-treatment of the untreated control group:<br>▪ Small (up to half SD)<br>▪ Moderate (half to one SD)<br>▪ Large (one to two SDs)<br>▪ Very large (over two SDs) |
| The minimal clinically important, large, and very large effects for the GRADE approach were defined as half, one, and two standard deviations of the response of the control (underage) group (Norman et al., 2003). |
| **References** |
| Norman GR, Sloan JA, Wyrwich KW. Interpretation of changes in health-related quality of life: the remarkable universality of half a standarddeviation. Med Care. 2003;41:582–92. |

**Supplementary Material S2.** Eligibility criteria for the inclusion of primary studies.

| | **Inclusion criteria** | **Exclusion criteria** |
|---|---|---|
| Patients | Patients of any age, gender, or ethnicity with Class III malocclusion due to a retrusive maxilla, protrusive mandible, or a combination of both scheduled to research orthopedic treatment | Patients with congenital malformations, clefts, any other syndromes, or obstructive sleep apnea |
| Intervention | Any kind of orthopedic treatment for Class III with facemask / reverse headgear or chin-cup | Any other kind of intervention |
| Control | Growing patients with skeletal Class III maloclussion without treatment | Untreated patients without Class III malocclusion |
| Outcome | Airway dimensions | - |
| Studies | Randomized and prospective / retrospective non-randomized controlled clinical trials | Animal studies, case reports/series, non-clinical studies, and cross-sectional studies |

**Supplementary Material S3.** Literature search (as of August 4th, 2020) for each database with the corresponding hits.

| Nr | Database | Search | Limits | |
|---|---|---|---|---|
| 1 | MEDLINE (through PubMed) | ((("Class III" OR "Cl. III" NOT "Class II" OR ((maxill* OR upper) AND retrognath*) OR ((mandib* OR lower) AND prognath*) OR (anter* AND (crossbite* OR "cross-bite" OR "cross-bites"))) AND (((orthop* OR orthodon* OR functional) AND (treatment* OR therap* OR appliance*)) OR facemask* OR Delaire OR "reverse headgear" OR "reverse Activator" OR chincup OR "chin-cup") AND (airway* OR ((pharyn* OR oropharyn* OR nasopharyn* OR hypopharyn*) AND (volume OR area OR cephalomet*))))) | | 253 |
| 3 | CDSR | Same as MEDLINE | | 1 |
| 4 | CENTRAL | Same as MEDLINE | | 34 |
| 5 | DARE | Same as MEDLINE | | 0 |
| 6 | Embase | Same as MEDLINE | | 120 |
| 7 | Scopus | ( TITLE-ABS-KEY ( ( "Class III" OR "Cl. III" OR ( ( maxill* OR upper ) AND retrognath* ) OR ( ( mandib* OR lower ) AND prognath* ) OR ( anter* AND ( crossbite* OR "cross-bite" OR "cross-bites" ) ) ) ) AND TITLE-ABS-KEY ( ( ( ( orthop* OR orthodon* OR functional ) AND ( treatment* OR therap* OR appliance* ) ) OR facemask* OR delaire OR "reverse headgear" OR "reverse Activator" OR chincup OR "chin-cup" ) ) AND TITLE-ABS-KEY ( ( airway* OR ( ( pharyn* OR oropharyn* OR nasopharyn* OR hypopharyn* ) AND ( volume OR area OR cephalomet* ) ) ) ) ) | Dentistry | 125 |
| 8 | WOK | Same as MEDLINE | Dentistry, oral surgery, medicine | 200 |
| 9 | VHL | Same as MEDLINE | | 116 |
| **Sum (with overlaps)** | | | | 849 |
| **Sum (without overlaps)** | | | | 539 |

CDSR, Cochrane Database of Systematic Reviews; DARE, Cochrane Database of Abstracts of Reviews of Effects; CENTRAL, Cochrane Central Register of Controlled Trials; VHL, virtual health library; WOK, Web of Knowledge.

**Supplementary Material S4**. List of studies identified from the literature search and their inclusion/exclusion status with reasons.

| Nr | Paper | Status |
|---|---|---|
| 1 | [No authors] [Lung transplantation in emphysema]. Medicina (B Aires). 1997;57(5):521-9. | Excluded by title |
| 2 | {IRCT138903033915N} Hemodynamic responses to intubation in hypertensive patients. Http://wwwwhoint/trialsearch/trial2aspx? Trialid=irct138903033915n2. 2011. | Excluded by title |
| 3 | {IRCT201108167346N} Effect of continuous positive airway pressure in the treatment of refractory hypertension in patients with sever obstructive sleep apnea. Http://wwwwhoint/trialsearch/trial2aspx? Trialid=irct201108167346n1. 2011. | Excluded by title |
| 4 | {NCT} Comparison of Treatment Effects of PowerScope2 and Forsus Using CBCT. Https://clinicaltrialsgov/show/nct03296644. 2017. | Excluded by title |
| 5 | Abdelrahman TE, Takahashi K, Tamura K, Nakao K, Hassanein KM, Alsuity A, et al. Impact of different surgery modalities to correct class III jaw deformities on the pharyngeal airway space. J Craniofac Surg. 2011;22(5):1598-601. | Excluded by title |
| 6 | Abreu MFFd. Análise dos aspectos biomédicos gerais e bucais em pacientes com doença de Alzheimer. 2019. p. 68-. | Excluded by title |
| 7 | Acevedo Pérez JL, Sánchez López A, Núñez Núñez C. Logopedia en paciente con mapeo cortical intraoperatorio. Rev logop foniatr audiol (Ed impr). 2017;37(1):43-9. | Excluded by title |
| 8 | Adams HR, Defendorf S, Vierhile A, Mink JW, Marshall FJ, Augustine EF. A novel, hybrid, single- and multi-site clinical trial design for CLN3 disease, an ultra-rare lysosomal storage disorder. Clinical trials (London, England). 2019;16(5):555-60. | Excluded by title |
| 9 | Adolfo JR, Dhein W, Sbruzzi G. Intensity of physical exercise and its effect on functional capacity in COPD: systematic review and meta-analysis. J bras pneumol. 2019;45(6):e20180011-e. | Excluded by title |
| 10 | Akarsu-Guven B, Karakaya J, Ozgur F, Aksu M. Growth-related changes of skeletal and upper-airway features in bilateral cleft lip and palate patients. Am J Orthod Dentofacial Orthop. 2015;148(4):576-86. | Excluded by title |
| 11 | Albino CC, Graf H, Paz-Filho G, Diehl LA, Olandoski M, Sabbag A, et al. Radioiodine plus recombinant human thyrotropin do not cause acute airway compression and are effective in reducing multinodular goiter. Braz j med biol res. 2010;43(3):303-9. | Excluded by title |
| 12 | Alcalde J, Pastor MJ, Quesada JL, Martín E, García Tapia R. Reconstrucción de defectos orofaríngeos con colgajo lateral de brazo. Acta otorrinolaringol esp. 2001;52(1):39-44. | Excluded by title |
| 13 | Al-Dohan AM, Al-Jewair TS. Limited Evidence Suggests That Presurgical Orthodontics May Not Be Needed for Orthognathic Surgery Patients. Journal of Evidence-Based Dental Practice. 2017;17(1):39-41. | Excluded by title |
| 14 | Alfwaress F, Al Maaitah E, Al-Khateeb S, Abu Zama Z. The relationship of vocal tract dimensions and substitution of the palatal approximant /j/ for the alveolar trill /r. International Journal of Speech-Language Pathology. 2015;17(5):518-26. | Excluded by title |
| 15 | Alkawari HM, Albalbeesi HO, Alhendi AA, Alhuwaish HA, Al Jobair AA, Baidas L. Pharyngeal airway dimensional changes after premolar extraction in skeletal class II and class III orthodontic patients. Journal of Orthodontic Science. 2018;7(1). | Excluded by title |
| 16 | Allareddy V, Ching N, Macklin EA, Voelz L, Weintraub G, Davidson E, et al. Craniofacial features as assessed by lateral cephalometric measurements in children with Down syndrome. Prog Orthod. 2016;17(1):35. | Excluded by title |
| 17 | Almeida RCC, Artese F, Carvalho FdAR, Cunha RD, Almeida MAdO. Comparação entre a radiografia de cavum e a cefalométrica de perfil na avaliação da nasofaringe e das adenoides por otorrinolaringologistas. Dental press j orthod (Impr). 2011;16(1):e1-e10. | Excluded by title |
| 18 | Al-Moraissi EA, Al-Magaleh SM, Iskandar RA, Al-Hendi EA. Impact on the pharyngeal airway space of different orthognathic procedures for the prognathic mandible. Int J Oral Maxillofac Surg. 2015;44(9):1110-8. | Excluded by title |
| 19 | Almuzian M, Almukhtar A, Ju X, Al-Hiyali A, Benington P, Ayoub A. Effects of Le Fort I Osteotomy on the Nasopharyngeal Airway-6-Month Follow-Up. J Oral Maxillofac Surg. 2016;74(2):380-91. | Excluded by title |
| 20 | Alonso-Rodriguez E, Gomez E, Martin M, Munoz JM, Hernandez-Godoy J, Burgueno M. Beckwith-Wiedemann Syndrome: Open bite evolution after tongue reduction. Med Oral Patol Oral Cir Bucal. 2018;23(2):e225-e9. | Excluded by title |
| 21 | Alves RdSA, Vianna FdAF, Pereira CAdC. Fenótipos clínicos de asma grave. J bras pneumol. 2008;34(9):646-53. | Excluded by title |
| 22 | Amini F, Borzabadi-Farahani A, Behnam-Roudsari G, Jafari A, Shahidinejad F. Assessment of the uvulo-glossopharyngeal dimensions in patients with beta-thalassemia major. Sleep Breath. 2013;17(3):943-9. | Excluded by title |
| 23 | Ancochea J, Gómez García T, Miguel Díez Jd. Hacia un tratamiento individualizado e integrado del paciente con EPOC. Arch bronconeumol (Ed impr). 2010;46(supl.10):14-8. | Excluded by title |
| 24 | Andrade CRd, Chatkin JM, Camargos PAM. Avaliação do grau de controle clínico, espirométrico e da intensidade do processo inflamatório na asma. J pediatr (Rio J). 2010;86(2):93-100. | Excluded by title |
| 25 | Andrea R, Lopez-Giraldo A, Falces C, Sobradillo P, Sanchis L, Gistau C, et al. Lung function abnormalities are highly frequent in patients with heart failure and preserved ejection fraction. Heart Lung Circ. 2014;23(3):273-9. | Excluded by title |
| 26 | Ankichetty S, Chung F. Considerations for patients with obstructive sleep apnea undergoing ambulatory surgery. Current Opinion in Anaesthesiology. 2011;24(6):605-11. | Excluded by title |
| 27 | Annoni R, Silva WR, Mariano MdS. Análise de parâmetros funcionais pulmonares e da qualidade de vida na revascularização do miocárdio. Fisioter mov. 2013;26(3):525-36. | Excluded by title |
| 28 | Antonia Rodriguez M, Friedberg JP, DiGiovanni A, Binhuan W, Wylie-Rosett J, Sangmin H, et al. A Tailored Behavioral Intervention to Promote Adherence to the DASH Diet. American journal of health behavior. 2019;43(4):659-70. | Excluded by title |
| 29 | Araújo CFdSNd, Braga PLdS, Ferreira JDB. Tratamento tardio de fratura condilar: Relato de caso. Rev cir traumatol buco-maxilo-fac. 2013;13(3):17-24. | Excluded by title |
| 30 | Athanasiou AE, Toutountzakis N, Mavreas D, Ritzau M, Wenzel A. Alterations of hyoid bone position and pharyngeal depth and their relationship after surgical correction of mandibular prognathism. Am J Orthod Dentofacial Orthop. 1991;100(3):259-65. | Excluded by title |
| 31 | Ávila Martínez RJ, Mariscal de Alba A, Zuluaga Bedoya M, Marrón Fernández C, Trujillo MD, Rivas C, et al. Traqueotomías abiertas a pie de cama. Rev patol respir. 2016;19(2):44-7. | Excluded by title |
| 32 | Azeredo Bittencourt L, Luz G, Guimaraes T, Silva L, Badke L, Millani A, et al. Effect of treatment of mild obstructive sleep apnea on quality of life, mood and sustained attention: Randomized, parallel, single-blind and controlled study. American Journal of Respiratory and Critical Care Medicine. 2018;197(MeetingAbstracts). | Excluded by title |
| 33 | Azevedo KRS. Teste de broncodilatação: a incorporação de novos parâmetros na sua avaliação. Pulmão RJ. 2015;24(1):8-13. | Excluded by title |
| 34 | Azevedo MS, Machado AW, Barbosa Ida S, Esteves LS, Rocha VA, Bittencourt MA. Evaluation of upper airways after bimaxillary orthognathic surgery in patients with skeletal Class III pattern using cone-beam computed tomography. Dental Press J Orthod. 2016;21(1):34-41. | Excluded by title |
| 35 | Bacher M, Linz A, Buchenau W, Arand J, Krimmel M, Poets C, et al. [Treatment of infants with Pierre Robin sequence]. Laryngorhinootologie. 2010;89(10):621-9. | Excluded by title |
| 36 | Baha A, Ekici B, Ogan N, Akpinar EE. A case of sjögren's syndrome-related pulmonary arterial hypertension treated with Iloprost and Bosentan combination therapy. Respiratory Case Reports. 2018;7(2):59-62. | Excluded by title |
| 37 | Bartzela TN. Treatment approaches to syndromes affecting craniofacial and dental structures. Journal of the World Federation of Orthodontists. 2019;8(4):131-7. | Excluded by title |
| 38 | Bassi E, Miranda LC, Tierno PFGMM, Ferreira CB, Cadamuro FM, Figueiredo VR, et al. Atendimento às vítimas de lesão inalatória por incêndio em ambiente fechado: o que aprendemos com a tragédia de Santa Maria. Rev bras ter intensiva. 2014;26(4):421-9. | Excluded by title |
| 39 | Baydur A. Recent developments in the physiological assessment of sarcoidosis: Clinical implications. Current Opinion in Pulmonary Medicine. 2012;18(5):499-505. | Excluded by title |
| 40 | Bedhet N, Mercier J, Gordeeff A, Mouzard A, Delaire J. [Labioglossopexy in Pierre Robin syndrome. Our experiences apropos of 70 cases]. Rev Stomatol Chir Maxillofac. 1990;91(5):326-34. | Excluded by title |
| 41 | Betancourt-Peña J, Tonguino-Rosero S. Impacto de la oxigenoterapia domiciliaria en la capacidad funcional de pacientes con enfermedad pulmonar obstructiva crónica. Rehabilitación (Madr, Ed impr). 2016;50(1):13-8. | Excluded by title |
| 42 | Bisinotto FMB, Seabra BC, Lóes FBP, Martins LB, Silveira LAMd. Postoperative angioedema induced by angiotensin-converting enzyme inhibitor: case report. Rev bras anestesiol. 2019;69(5):521-6. | Excluded by title |

| | | |
|---|---|---|
| 43 | Bitter N, Roeg D, Van Nieuwenhuizen C, Van Weeghel J. Training professionals in a recovery-oriented methodology: a mixed method evaluation. Scandinavian journal of caring sciences. 2019;33(2):457-66. | Excluded by title |
| 44 | Bjorklund KA, Billmire DA. Mandibular Body Resection and Setback for Severe Malocclusion in Lymphatic Malformations. J Craniofac Surg. 2016;27(3):724-6. | Excluded by title |
| 45 | Blanco Pérez JJ, Zamarrón Sanz C, Almazán Ortega R, García García M, López Castro J, Tumbeiro Novoa M. Síndrome de apnea del sueño en la insuficiencia cardiaca. Efecto de la presión positiva continua en la vía aérea. An med interna (Madr, 1983). 2008;25(1):15-9. | Excluded by title |
| 46 | Borborema dos Santos VD, Assis GMd, Pereira da Silva JS, Rocha Germano A. Glosectomía parcial en paciente portador del síndrome de Beckwith-Wiedemann: relato del caso. Rev esp cir oral maxilofac. 2015;37(4):202-6. | Excluded by title |
| 47 | Borborema dos Santos VD, de Assis GM, da Silva JSP, Germano AR. Partial glossectomy in a patient carrier of Beckwith-Wiedemann syndrome: Presentation of a case. Revista Espanola de Cirugia Oral y Maxilofacial. 2015;37(4):202-6. | Excluded by title |
| 48 | Brogan WF. The stability of maxillary expansion. Aust Dent J. 1977;22(2):92-9. | Excluded by title |
| 49 | Bronfman CN. Avaliação das vias aéreas superiores por meio de tomografia computadorizada Cone-beam em pacientes Classe III submetidos à cirurgia bimaxilar. 2016:104-. | Excluded by title |
| 50 | Brunetto DP, Velasco L, Koerich L, Araujo MTD. Prediction of 3-dimensional pharyngeal airway changes after orthognathic surgery: A preliminary study. American Journal of Orthodontics and Dentofacial Orthopedics. 2014;146(3):299-309. | Excluded by title |
| 51 | Bruno LP, Motta JPS. Tratamento Endoscópico com Válvulas Endobrônquicas nos Pacientes com Enfisema Pulmonar. Pulmão RJ. 2017;26(1):39-44. | Excluded by title |
| 52 | Buchenau W, Wenzel S, Bacher M, Muller-Hagedorn S, Arand J, Poets CF. Functional treatment of airway obstruction and feeding problems in infants with Robin sequence. Arch Dis Child Fetal Neonatal Ed. 2017;102(2):F142-f6. | Excluded by title |
| 53 | Burgos MA, Sevilla García MA, Sanmiguel Rojas E, Pino Cd, Fernández Velez C, Piqueras F, et al. Cirugía virtual para pacientes con obstrucción nasal: empleo de un software basado en dinámica de fluidos (MeComLand(R), Digbody(R) & Noseland(R)) para documentar parámetros objetivos de flujo y optimizar resultados quirúrgicos. Acta otorrinolaringol esp. 2018;69(3):125-33. | Excluded by title |
| 54 | Bussoni MF, Guirado GN, Matsubara LS, Roscani MG, Polegato BF, Minamoto ST, et al. Diastolic function and functional capacity after a single session of continuous positive airway pressure in patients with compensated heart failure. Clinics. 2014;69(5):354-9. | Excluded by title |
| 55 | Butković D. Some pediatric syndromes with difficult airways in anesthesia induction. Acta Medica Croatica. 2018;72:47-56. | Excluded by title |
| 56 | C GPd, Saldías P F. Entrenamiento muscular inspiratorio en el paciente con enfermedad pulmonar obstructiva crónica. Rev chil enferm respir. 2011;27(2):116-23. | Excluded by title |
| 57 | Cabedo García VR, Rodrigo Garcés Asemany C, Cortes Berti A, Oteo Elso JT, Ballester Salvador JB. Eficacia de la utilización correcta de los dispositivos de inhalación en pacientes con enfermedad pulmonar obstructiva crónica: ensayo clínico aleatorizado. Med clín (Ed impr). 2010;135(13):586-91. | Excluded by title |
| 58 | Cabral DMG, Abrahão Júnior LJ, Marques CHD, Pereira BdB, Pedrosa RC. Disfagia orofaríngea na doença de Chagas crônica: avaliação fonoaudiológica, videofluoroscópica e esofagomanométrica. Acta fisiátrica. 2015;22(1). | Excluded by title |
| 59 | Cadenat H, Boutault F. Strategy of the Decision in Orthognathic Surgery Part 1. Importance of the Functional Factors for Choosing the Surgical Procedure Theoretical Study. Revue de Stomatologie et de Chirurgie Maxillo-Faciale. 1992;93(1):25-31. | Excluded by title |
| 60 | Caillot A, Ambroise B, Benateau H, Veyssiere A. Impact of early intravelar veloplasty at six months on mandibular growth in patients with Pierre Robin Sequence. J Craniomaxillofac Surg. 2018;46(7):1059-64. | Excluded by title |
| 61 | Cakirer B, Kucukkeles N, Nevzatoglu S, Koldas T. Sagittal airway changes: rapid palatal expansion versus Le Fort I osteotomy during maxillary protraction. Eur J Orthod. 2012;34(3):381-9. | Excluded by title |
| 62 | Calabrese C, Corcione N, Rea G, Stefanelli F, Meoli I, Vatrella A. Impact of long-term treatment with inhaled corticosteroids and bronchodilators on lung function in a patient with post-infectious bronchiolitis obliterans. J bras pneumol. 2016;42(3):228-31. | Excluded by title |
| 63 | Call Mañosa S, Pujol Garcia A, Chacón Jordan E, Martí Hereu L, Pérez Tejero G, Gómez Simón V, et al. Plan de cuidados individualizado durante oxigenación con membrana extracorpórea. Caso clinic. Enferm intensiva (Ed impr). 2016;27(2):75-80. | Excluded by title |
| 64 | Capan E, Ersu R, Kiyan E, Yener HM, Arman A, Kilicoglu H. Monoblock appliance for treatment of children with sleep disordered breathing. American Journal of Respiratory and Critical Care Medicine. 2015;191. | Excluded by title |
| 65 | Capan E, Kiyan E, Ersu R, Kilicoglu H. Monoblock appliance in children with obstructive sleep apnea is an effective treatment modality. European Respiratory Journal. 2014;44. | Excluded by title |
| 66 | Caprioglio A, Zucconi M, Calori G, Troiani V. Habitual snoring, OSA and craniofacial modification. Orthodontic clinical and diagnostic aspects in a case control study. Minerva Stomatol. 1999;48(4):125-37. | Excluded by title |
| 67 | Carlos-Villafranca Fd, Cobo-Plana J, Macías-Escalada E, Martínez J. Vía aérea difícil: interacciones entre ortodoncia y anestesiología. RCOE, Rev Ilustre Cons Gen Col Odontól Estomatól Esp. 2005;10(2):187-95. | Excluded by title |
| 68 | Carra MC, Lavigne G, Rompré P. Sleep bruxismand headache in adolescents. Sleep Medicine. 2011;12:S21. | Excluded by title |
| 69 | Casanueva FL, Alzérreca JA. Válvula nasal en rinoplastía. Rev otorrinolaringol cir cabeza cuello. 2017;77(4):441-8. | Excluded by title |
| 70 | Castrillo Tambay M, Zubillaga Rodríguez I, Sánchez Aniceto G, Gutiérrez Díaz R, Gutiérrez Díez M, Montalvo Moreno JJ. Distracción osteogénica mandibular en microrretrognatia severa del adulto. Rev esp cir oral maxilofac. 2005;27(4):231-7. | Excluded by title |
| 71 | Catalán Escudero P, Uriarte Valiente M, Morató Robert P, Souto Romero H, Olavi IP, Martínez García E. Manejo anestésico para la resección de masa mediastínica anterior (MMA) en paciente pediátrico. Descripción de un caso clínico. Rev esp anestesiol reanim. 2020;67(1):39-43. | Excluded by title |
| 72 | Chang HP, Liu PH, Chang HF, Chang CH. Thin-plate spline (TPS) graphical analysis of the mandible on cephalometric radiographs. Dentomaxillofac Radiol. 2002;31(2):137-41. | Excluded by title |
| 73 | Chemtob K, Rocchi M, Arbour-Nicitopoulos K, Kairy D, Fillion B, Sweet SN. Using tele-health to enhance motivation, leisure time physical activity, and quality of life in adults with spinal cord injury: a self-determination theory-based pilot randomized control trial. Psychology of sport and exercise. 2019;43:243-52. | Excluded by title |
| 74 | Chen F, Terada K, Hanada K, Saito I. Predicting the pharyngeal airway space after mandibular setback surgery. J Oral Maxillofac Surg. 2005;63(10):1509-14. | Excluded by title |
| 75 | Chen F, Terada K, Hua Y, Saito I. Effects of bimaxillary surgery and mandibular setback surgery on pharyngeal airway measurements in patients with Class III skeletal deformities. Am J Orthod Dentofacial Orthop. 2007;131(3):372-7. | Excluded by title |
| 76 | Chen H, Yagi K, Tsuda H, Almeida F, Lowe A. Klearwa yTM oral appliances for pediatric patients with retruded mandibles. Canadian Respiratory Journal. 2012;19(3):e39. | Excluded by title |
| 77 | Chen Q, Zhao Y, Qian Y, Lu C, Shen G, Dai J. A genetic-phenotypic classification for syndromic micrognathia. J Hum Genet. 2019;64(9):875-83. | Excluded by title |
| 78 | Choi JW, Park YJ, Lee CY. Posterior Pharyngeal Airway in Clockwise Rotation of Maxillomandibular Complex Using Surgery-first Orthognathic Approach. Plast Reconstr Surg Glob Open. 2015;3(8):e485. | Excluded by title |
| 79 | Choi SH, Kang DY, Kim YH, Hwang CJ. Severe skeletal Class III malocclusion treated with 2-stage orthognathic surgery with a mandibular step osteotomy. Am J Orthod Dentofacial Orthop. 2014;145(4 Suppl):S125-35. | Excluded by title |
| 80 | Chouard CH. Did Napoleon suffer from chronic rhonchopathy? Acta Oto-Laryngologica. 2017;137(4):361-4. | Excluded by title |
| 81 | Cifuentes J, Palisson F, Valladares S, Jerez D. Life-threatening complications following orthognathic surgery in a patient with undiagnosed hereditary angioedema. J Oral Maxillofac Surg. 2013;71(4):e185-8. | Excluded by title |
| 82 | Cifuentes J, Valladares S, Barrera A, Jerez D. Life threatening complication posterior to orthognathic surgery in a patient with an undiagnosed hereditary angioedema. International Journal of Oral and Maxillofacial Surgery. 2011;40(10):1196. | Excluded by title |
| 83 | Clark HR, Powell AB, Simmons KA, Ayubi T, Kale SD. Endocytic Markers Associated with the Internalization and Processing of Aspergillus fumigatus Conidia by BEAS-2B Cells. mSphere. 2019;4(1). | Excluded by title |
| 84 | Cobb ARM, Green B, Gill D, Ayliffe P, Lloyd TW, Bulstrode N, et al. The surgical management of Treacher Collins syndrome. British Journal of Oral and Maxillofacial Surgery. 2014;52(7):581-9. | Excluded by title |
| 85 | Cortes M, Gomez M, Park S, Singh D. A combined approach for upper airway remodeling for skeletal class iii malocclusion with complex OSA. Sleep. 2018;41:A206. | Excluded by title |
| 86 | Costa Solà R, Muñoz Gall X, Avilés Huertas B, Drobnic Martínez ME, Orriols Martínez R. Síndrome de disfunción reactiva de las vías | Excluded by title |

| | | |
|---|---|---|
| | respiratorias. Estudio de 18 casos. Med clín (Ed impr). 2005;124(11):419-22. | |
| 87 | Crisanti AS, Murray-Krezan C, Reno J, Killough C. Effectiveness of Peer-Delivered Trauma Treatment in a Rural Community: a Randomized Non-inferiority Trial. Community mental health journal. 2019;55(7):1125-34. | Excluded by title |
| 88 | Cui Y, Moriyama M, Chayama K, Liu Y, Ya C, Muzembo BA, et al. Efficacy of a self-management program in patients with chronic viral hepatitis in China. BMC nursing. 2019;18(1):N.PAG. | Excluded by title |
| 89 | Cunha MS, Andrade V, Guedes CAV, Meneghetti CHZ, Aguiar AP, Cardoso AL. Assessment of functional capacity and quality of life in chronic renal patients under hemodialysis treatment. Fisioterapia e pesquisa. 2009;16(2):155-60. | Excluded by title |
| 90 | Dahy K, Takahashi K, Saito K, Kiso H, Rezk I, Oga T, et al. Gender differences in morphological and functional outcomes after mandibular setback surgery. J Craniomaxillofac Surg. 2018;46(6):887-92. | Excluded by title |
| 91 | Danaei SM, Ajami S, Etemadi H, Azadeh N. Assessment of the effect of maxillary protraction appliance on pharyngeal airway dimensions in relation to changes in tongue posture. Dent Res J (Isfahan). 2018;15(3):208-14. | Excluded by title |
| 92 | Davies SF, McQuaid KR, Iber C, McArthur CD, Path MJ, Beebe DS, et al. Extreme dyspnea from unilateral pulmonary venous obstruction. Demonstration of a vagal mechanism and relief by right vagotomy. American Review of Respiratory Disease. 1987;136(1):184-8. | Excluded by title |
| 93 | Dawjee SM. A simple appliance for the management of obstructive sleep apnoea--the MEDUNSA Anti-Snoring Device (MASD). Sadj. 2004;59(4):151-3. | Excluded by title |
| 94 | de Araújo-Filho AA, de Cerqueira-Neto ML, de Assis Pereira Cacau L, Oliveira GU, Cerqueira TCF, de Santana-Filho VJ. Effect of prophylactic non-invasive mechanical ventilation on functional capacity after heart valve replacement: a clinical trial. Clinics. 2017;72(10):618-23. | Excluded by title |
| 95 | de Jong EEC, Sanders KJC, Deist TM, van Elmpt W, Jochems A, van Timmeren JE, et al. Can radiomics help to predict skeletal muscle response to chemotherapy in stage IV non-small cell lung cancer? European journal of cancer. 2019;120:107-13. | Excluded by title |
| 96 | Denolf PL, Vanderveken OM, Marklund ME, Braem MJ. The status of cephalometry in the prediction of non-CPAP treatment outcome in obstructive sleep apnea patients. Sleep Med Rev. 2016;27:56-73. | Excluded by title |
| 97 | Detsky ME, Jivraj N, Adhikari NK, Friedrich JO, Pinto R, Simel DL, et al. Will This Patient Be Difficult to Intubate? The Rational Clinical Examination Systematic Review. Jama-Journal of the American Medical Association. 2019;321(5):493-503. | Excluded by title |
| 98 | DeWood C, Grimes M, Vaden JL. Is the benefit of early orthodontic treatment worth the burden? The Journal of the Tennessee Dental Association. 2006;86(2):12-7. | Excluded by title |
| 99 | Do JBA, Bellerive A, Julien A-S, Leclerc JE. Cleft Palates and Occlusal Outcomes in Pierre Robin Sequence. Otolaryngology-Head and Neck Surgery. 2019;160(2):246-54. | Excluded by title |
| 100 | D'Onofrio L. Oral dysfunction as a cause of malocclusion. Orthodontics & Craniofacial Research. 2019;22:43-8. | Excluded by title |
| 101 | Douglass J, O'Donnell O, Leslie R, Nienstadt L, DiGiorgio MJ. Increasing Patient Accessibility and Use of Hand Sanitizer Through Introduction of a Single-Dose Packet...46th Annual Conference, APIC 2019, Philadelphia, PA. American journal of infection control. 2019;47:S45. | Excluded by title |
| 102 | Dubé MP, Chan ES, Lake JE, Williams B, Kinslow J, Landay A, et al. A Randomized, Double-blinded, Placebo-controlled Trial of Sitagliptin for Reducing Inflammation and Immune Activation in Treated and Suppressed Human Immunodeficiency Virus Infection. Clinical infectious diseases. 2019;69(7):1165-72. | Excluded by title |
| 103 | Durán-Cantolla J, Cancelo Díaz L, Álvarez Ruiz-Larrinaga A. EPOC y apneas del sueño. Arch bronconeumol (Ed impr). 2010;46(supl.3):2-10. | Excluded by title |
| 104 | Echarri Lobiondo P, Pérez Campoy MA, Echarri J. El paciente ortodóncico en dentición mixta y los trastornos del sueño. Rev Ateneo Argent Odontol. 2019;61(2):13-25. | Excluded by title |
| 105 | Edwards PD, Rahbar R, Ferraro NF, Burrows PE, Mulliken JB. Lymphatic malformation of the lingual base and oral floor. Plast Reconstr Surg. 2005;115(7):1906-15. | Excluded by title |
| 106 | Efendiyeva R, Aydemir H, Karasu H, Toygar-Memikoglu U. Pharyngeal airway space, hyoid bone position, and head posture after bimaxillary orthognathic surgery in Class III patients: long-term evaluation. Angle Orthod. 2014;84(5):773-81. | Excluded by title |
| 107 | El Aouame A, Daoui A, El Quars F. Nasal breathing and the vertical dimension: A cephalometric study. Int Orthod. 2016;14(4):491-502. | Excluded by title |
| 108 | El-Bialy TH, Razdolsky Y, Kravitz ND, Dessner S, Elgazzar RF. Long-term results of bilateral mandibular distraction osteogenesis using an intraoral tooth-borne device in adult Class II patients. International Journal of Oral and Maxillofacial Surgery. 2013;42(11):1446-53. | Excluded by title |
| 109 | Elborn JS. Key advances along the CF pipeline. Pediatric Pulmonology. 2011;46:179-80. | Excluded by title |
| 110 | Enacar A, Aksoy AU, Sencift Y, Haydar B, Aras K. Changes in hypopharyngeal airway space and in tongue and hyoid bone positions following the surgical correction of mandibular prognathism. Int J Adult Orthodon Orthognath Surg. 1994;9(4):285-90. | Excluded by title |
| 111 | Engel M, Berger M, Hoffmann J, Kuhle R, Ruckschloss T, Ristow O, et al. Midface correction in patients with Crouzon syndrome is Le Fort III distraction osteogenesis with a rigid external distraction device the gold standard? J Craniomaxillofac Surg. 2019;47(3):420-30. | Excluded by title |
| 112 | Favaloro RR, Machain A, Gómez C, Perrone SV, Klein F, Presa C, et al. Lung transplantation for emphysema. Medicina. 1997;57(5):521-9. | Excluded by title |
| 113 | Fernandes ADO, Sens Y, Xavier VB, Miorin LA, Alves V. Functional and Respiratory Capacity of Patients with Chronic Kidney Disease Undergoing Cycle Ergometer Training during Hemodialysis Sessions: a Randomized Clinical Trial. International journal of nephrology. 2019:1-7. | Excluded by title |
| 114 | Fernandes SCdS, Santos RSd, Giovanetti EA, Taniguchi C, Silva CSdM, Eid RAC, et al. Impact of respiratory therapy in vital capacity and functionality of patients undergoing abdominal surgery. Einstein (São Paulo). 2016;14(2):202-7. | Excluded by title |
| 115 | Fielbaum C Ó. Avances en fibrosis quística. Rev Méd Clín Condes. 2011;22(2):150-9. | Excluded by title |
| 116 | Figueiró-Filho EA, Somensi RS, Queiroz GTd, Maia MZ, Miranda RCF. Ressonância nuclear magnética fetal: aplicabilidade e indicações no período pré-natal. Femina. 2009;37(2):63-9. | Excluded by title |
| 117 | Figueroa Casas JC, Schiavi E, Mazzei JA, López AM, Rhodius E, Ciruzzi J, et al. Recomendaciones para la prevención, diagnóstico y tratamiento de LA EPOC en la Argentina. Medicina (BAires). 2012;72(4,supl.1):1-33. | Excluded by title |
| 118 | FK93N RBR. A mode of mechanical ventilation applied without artificial airway in the post operative obesity surgery. Http://wwwwhoint/trialsearch/trial2aspx? Trialid=rbr-6fk93n. 2017. | Excluded by title |
| 119 | Flanagan D. A dual-laminate snore reduction appliance: a report of four cases. Gen Dent. 2010;58(4):e156-61. | Excluded by title |
| 120 | Freng A, Kvam E. Facial sagittal growth following partial, basal resection of the nasal septum: A retrospective study in man. European Journal of Orthodontics. 1979;1(2):89-96. | Excluded by title |
| 121 | Gahona O, Argandona J, Pizarro A, Diaz V. Impacto de las prótesis removibles en la posición lingual, patrón de deglución y calibre sagital de la vía aérea orofaríngea. Rev Fac Odontol Univ Antioq. 2013;24(2):243-57. | Excluded by title |
| 122 | Gandedkar NH, Chng CK, Por YC, Yeow VKL, Ow ATC, Seah TE. Influence of Bimaxillary Surgery on Pharyngeal Airway in Class III Deformities and Effect on Sleep Apnea: A STOP-BANG Questionnaire and Cone-Beam Computed Tomography Study. J Oral Maxillofac Surg. 2017;75(11):2411-21. | Excluded by title |
| 123 | García García S, Carazo Fernández L, García JJ, Naveiro Rilo JC. Enfermedad pulmonar obstructiva crónica: los pacientes en la vida real. Estudio LEONPOC. Aten prim (Barc, Ed impr). 2017;49(10):603-10. | Excluded by title |
| 124 | García Menéndez M, Cuspineda Bravo E, Valiente Zaldívar C. Síndrome de apnea hipopnea del sueño: rol protagónico del ortodontista. Rev habanera cienc méd. 2014;13(2):207-18. | Excluded by title |
| 125 | Giuca MR, Pasini M, Galli V, Casani AP, Marchetti E, Marzo G. Correlations between transversal discrepancies of the upper maxilla and oral breathing. Eur J Paediatr Dent. 2009;10(1):23-8. | Excluded by title |
| 126 | Gogas HJ, Flaherty KT, Dummer R, Ascierto PA, Arance A, Mandala M, et al. Adverse events associated with encorafenib plus binimetinib in the COLUMBUS study: incidence, course and management. European journal of cancer. 2019;119:97-106. | Excluded by title |
| 127 | Gokce SM, Gorgulu S, Gokce HS, Bengi AO, Karacayli U, Ors F. Evaluation of pharyngeal airway space changes after bimaxillary orthognathic surgery with a 3-dimensional simulation and modeling program. Am J Orthod Dentofacial Orthop. 2014;146(4):477-92. | Excluded by title |
| 128 | Gokce SM, Gorgulu S, Karacayli U, Gokce HS, Battal B. Three-dimensional evaluation of nasal and pharyngeal airway after Le Fort I maxillary distraction osteogenesis. Int J Oral Maxillofac Surg. 2015;44(4):455-61. | Excluded by title |
| 129 | Gómez Candela C, Cantón Blanco A, Luengo Pérez LM, Olveira Fuster G. Eficacia, coste-efectividad y efectos sobre la calidad de vida de la suplementación nutricional. Nutr hosp. 2010;25(5):781-92. | Excluded by title |
| 130 | Gómez Tejada RA, Enghelmayer JI, Mosquera RP, Cánepa A, Luna C. Bronquiectasias no relacionadas a fibrosis quística en un hospital universitario: Relación entre compromiso funcional y extensión anatómica. Rev am med respir. 2014;14(4):365-74. | Excluded by title |

| | | |
|---|---|---|
| 131 | Goncalves JR, Buschang PH, Goncalves DG, Wolford LM. Postsurgical stability of oropharyngeal airway changes following counter-clockwise maxillo-mandibular advancement surgery. J Oral Maxillofac Surg. 2006;64(5):755-62. | Excluded by title |
| 132 | González Antolín O. Tratamiento local de un faringostoma secundario a osteorradionecrosis. Enferm clín (Ed impr). 2017;27(3):193-202. | Excluded by title |
| 133 | González MB, Casellas JB, Fernández Mondragón MP, Nuño VC, Amezaga JA, De Carlos Villafra F. Clinical, esthetic, and quality of life outcomes after telegnathic surgery in Caucasian OSAS patients. Cranio. 2020:1-8. | Excluded by title |
| 134 | Gopal R, Tripathi T, Rai P, Kanase A. Three-dimensional assessment of pharyngeal airway space by MRI in class II division 1 patients treated by twin block appliance. Journal of Clinical and Diagnostic Research. 2018;12(9):ZC20-ZC3. | Excluded by title |
| 135 | Grippaudo C, Paolantonio EG, Antonini G, Saulle R, La Torre G, Deli R. Association between oral habits, mouth breathing and malocclusion. Acta Otorhinolaryngol Ital. 2016;36(5):386-94. | Excluded by title |
| 136 | Guilleminault C, Stoohs R. Obstructive sleep apnea syndrome in children. Pediatrician. 1990;17(1):46-51. | Excluded by title |
| 137 | Hajek R, Russell SD, Lyon A, Lenihan D, Moreau P, Joshua D, et al. A substudy of the phase 3 endeavor study: serial echocardiographic assessment of patients with relapsed multiple myeloma (RMM) receiving carfilzomib plus dexamethasone or bortezomib plus dexamethasone. Haematologica. 2016;101:263-. | Excluded by title |
| 138 | Halder N, Choudhuri A, Gupta A, Raychowdhury R, Sarkar A, Pal R, et al. Pierre robin sequence (PRS) prenatal diagnostic limitation leading to management challenges after birth. Journal of Perinatal Medicine. 2019;47:eA316. | Excluded by title |
| 139 | Hall AB, Ziadi MC, Leech J, Chen SY, Burwash I, Renaud J, et al. Determination of the impact of short term continuous positive airway pressure on myocardial energetics in a randomized study of patients with chronic stable heart failure and obstructive sleep apnea. Circulation. 2011;124(21 Suppl 1):A14320. | Excluded by title |
| 140 | Halonen K, Hukki J, Arte S, Hurmerinta K. Craniofacial structures and dental development in three patients with Nager syndrome. Journal of Craniofacial Surgery. 2006;17(6):1180-7. | Excluded by title |
| 141 | Hamada T, Ono T, Otsuka R, Honda E, Harada K, Kurabayashi T, et al. Mandibular distraction osteogenesis in a skeletal Class II patient with obstructive sleep apnea. American Journal of Orthodontics and Dentofacial Orthopedics. 2007;131(3):415-25. | Excluded by title |
| 142 | Harari D, Redlich M, Miri S, Hamud T, Gross M. The Effect of Mouth Breathing Versus Nasal Breathing on Dentofacial and Craniofacial Development in Orthodontic Patients. Laryngoscope. 2010;120(10):2089-93. | Excluded by title |
| 143 | Harris DJ, Wilson MR, Buckingham G, Vine SJ. No effect of transcranial direct current stimulation of frontal, motor or visual cortex on performance of a self-paced visuomotor skill. Psychology of sport and exercise. 2019;43:368-73. | Excluded by title |
| 144 | Heliovaara A, Ranta R, Rautio F. Craniofacial cephalometric morphology in six-year-old girls with submucous cleft palate and isolated cleft palate. Acta Odontologica Scandinavica. 2003;61(6):363-6. | Excluded by title |
| 145 | Hellquist R, Ponten B. The influence of infant periosteoplasty on facial growth and dental occlusion from five to eight years of age in cases of complete unilateral cleft lip and palate. Scandinavian Journal of Plastic and Reconstructive Surgery. 1979;13(2):305-12. | Excluded by title |
| 146 | Hermann NV, Darvann TA, Jensen BL, Dahl E, Bolund S, Kreiborg S. Early craniofacial morphology and growth in children with bilateral complete cleft lip and palate. Cleft Palate-Craniofacial Journal. 2004;41(4):424-38. | Excluded by title |
| 147 | Herrejón A, Palop J, Inchaurraga I, López A, Bañuls C, Hernández A, et al. Dosis bajas de acetato de megestrol aumentan el peso y mejoran la nutrición de los pacientes con enfermedad pulmonar obstructiva crónica grave y pérdida de peso. Med clín (Ed impr). 2011;137(5):193-8. | Excluded by title |
| 148 | Hetland A, K HH, Olseng M, Gjesdal O, Ross S, Saberniak J, et al. Three months treatment with adaptive servo-ventilation improves cardiac function and physical activity in patients with chronic heart failure and cheyne-stokes respiration in a prospective randomized controlled trial. Circulation. 2011;124(21). | Excluded by title |
| 149 | Hevel DJ, Amorose AJ, Lagally KM, Rinaldi-Miles A, Pierce S. Testing the effects of messaging on physical activity motivation in active and non-active adults. Psychology of sport and exercise. 2019;43:333-42. | Excluded by title |
| 150 | Hikita R, Kobayashi Y, Tsuji M, Kawamoto T, Moriyamad K. Long-term orthodontic and surgical treatment and stability of a patient with Beckwith-Wiedemann syndrome. American Journal of Orthodontics and Dentofacial Orthopedics. 2014;145(5):672-84. | Excluded by title |
| 151 | Hillier SL, Ferrieri P, Edwards MS, Ewell M, Ferris D, Fine P, et al. Phase 2, Randomized, Control Trial of Group B Streptococcus (GBS) Type III Capsular Polysaccharide-tetanus Toxoid (GBS III-TT) Vaccine to Prevent Vaginal Colonization With GBS III. Clinical infectious diseases. 2019;68(12):2079-86. | Excluded by title |
| 152 | Hochban W. Surgical treatment of obstructive sleep apnea. Oto-Rhino-Laryngologia Nova. 2000;10(3-4):149-61. | Excluded by title |
| 153 | Hoffstein V, Wright S. Improvement in upper airway structure and function in a snoring patient following orthognathic surgery. Journal of Oral and Maxillofacial Surgery. 1991;49(6):656-8. | Excluded by title |
| 154 | Hohenberger P, Bonvalot S, van Coevorden F, Rutkowski P, Stoeckle E, Olungu C, et al. Quality of surgery and surgical reporting for patients with primary gastrointestinal stromal tumours participating in the EORTC STBSG 62024 adjuvant imatinib study. European journal of cancer. 2019;120:47-53. | Excluded by title |
| 155 | Hsieh YJ, Chen YC, Chen YA, Liao YF, Chen YR. Effect of bimaxillary rotational setback surgery on upper airway structure in skeletal class III deformities. Plast Reconstr Surg. 2015;135(2):361e-9e. | Excluded by title |
| 156 | Hueto J, Cebollero P, Pascal I, Cascantea JA, Eguía VM, Teruel F, et al. La espirometría en atención primaria en Navarra. Arch bronconeumol (Ed impr). 2006;42(7):326-31. | Excluded by title |
| 157 | Hwang S, Chung CJ, Choi YJ, Huh JK, Kim KH. Changes of hyoid, tongue and pharyngeal airway after mandibular setback surgery by intraoral vertical ramus osteotomy. Angle Orthod. 2010;80(2):302-8. | Excluded by title |
| 158 | Hylton JB, Leon-Salazar V, Anderson GC, De Felippe NL. Multidisciplinary treatment approach in Treacher Collins syndrome. J Dent Child (Chic). 2012;79(1):15-21. | Excluded by title |
| 159 | Iannetti G, Polimeni A, Pagnoni M, Fadda MT, Ramieri V, Tecco S, et al. Upper airway volume after Le Fort III advancement in subjects with craniofacial malformation. J Craniofac Surg. 2011;22(1):351-5. | Excluded by title |
| 160 | Ikavalko T, Narhi M, Eloranta AM, Lintu N, Myllykangas R, Vierola A, et al. Predictors of sleep disordered breathing in children: the PANIC study. Eur J Orthod. 2018;40(3):268-72. | Excluded by title |
| 161 | Ike D, Jamami M, Marino DM, Ruas G, Pessoa BV, Lorenzo VAPD. Efeitos do exercício resistido de membros superiores na força muscular periférica e na capacidade funcional do paciente com DPOC. Fisioter mov. 2010;23(3):429-37. | Excluded by title |
| 162 | Irani SK, Oliver DR, Movahed R, Kim YI, Thiesen G, Kim KB. Pharyngeal airway evaluation after isolated mandibular setback surgery using cone-beam computed tomography. Am J Orthod Dentofacial Orthop. 2018;153(1):46-53. | Excluded by title |
| 163 | Ishida T, Manabe A, Yang SS, Watakabe K, Abe Y, Ono T. An orthodontic-orthognathic patient with obstructive sleep apnea treated with Le Fort I osteotomy advancement and alar cinch suture combined with a muco-musculo-periosteal V-Y closure to minimize nose deformity. Angle Orthod. 2019. | Excluded by title |
| 164 | Izquierdo Alonso JL, Rodríguez González-Moro JM, Lucas Ramos Pd, Martín Centeno A, Gobartt Vázquez E. ¿Ha cambiado el manejo de la EPOC en España? Resultados de un estudio multicéntrico comunitario (VICE). Rev clín esp (Ed impr). 2008;208(1):18-25. | Excluded by title |
| 165 | Jakobsone G, Stenvik A, Espeland L. The effect of maxillary advancement and impaction on the upper airway after bimaxillary surgery to correct Class III malocclusion. Am J Orthod Dentofacial Orthop. 2011;139(4 Suppl):e369-76. | Excluded by title |
| 166 | Janssens J, Weber C, Herrmann F, Cantero C, Pessina A, Matis C, et al. Can Early Introduction of Palliative Care Limit Intensive Care, Emergency and Hospital Admissions in Patients with Severe Chronic Obstructive Pulmonary Disease? A Pilot Randomized Study. Respiration; international review of thoracic diseases. 2019;97(5):406-15. | Excluded by title |
| 167 | Jena AK, Singh SP, Utreja AK. Effectiveness of twin-block and Mandibular Protraction Appliance-IV in the improvement of pharyngeal airway passage dimensions in Class II malocclusion subjects with a retrognathic mandible. Angle Orthod. 2013;83(4):728-34. | Excluded by title |
| 168 | Jiang C, Yi Y, Jiang C, Fang S, Wang J. Pharyngeal Airway Space and Hyoid Bone Positioning After Different Orthognathic Surgeries in Skeletal Class II Patients. Journal of Oral and Maxillofacial Surgery. 2017;75(7):1482-90. | Excluded by title |
| 169 | Jiang YY, Xu X, Su HL, Liu DX. Gender-related difference in the upper airway dimensions and hyoid bone position in Chinese Han children and adolescents aged 6-18 years using cone beam computed tomography. Acta Odontologica Scandinavica. 2015;73(5):391-400. | Excluded by title |
| 170 | Jicha GA, Bardach SH, Schmitt FA, Fardo DW, Kryscio RJ, Abner EL, et al. Visual Arts Education improves self-esteem for persons with dementia and reduces caregiver burden: a randomized controlled trial. Dementia (14713012). 2019;18(7/8):3130-42. | Excluded by title |

| | | |
|---|---|---|
| 171 | Jing T, Zhenghuan S, Qingming B, Pengyi L, Lianbing G. Effects of volume-controlled ventilation vs. pressure-controlled ventilation on respiratory function and inflammatory factors in patients undergoing video-assisted thoracoscopic radical resection of pulmonary carcinoma. Journal of thoracic disease. 2018;10(3):1483-9. | Excluded by title |
| 172 | Jun T, Wei H, Xuhui C, Qian L, Tingwei W, Hao J, et al. Liuzijue Qigong: a Voice Training Method For Unilateral Vocal Fold Paralysis Patients. Annals of otology, rhinology, and laryngology. 2019;128(7):654-61. | Excluded by title |
| 173 | Jung YW, On SW, Chung KR, Song SI. Simultaneous Glossectomy with Orthognathic Surgery for Mandibular Prognathism. Maxillofac Plast Reconstr Surg. 2014;36(5):214-8. | Excluded by title |
| 174 | Kaditis AG, Alvarez MLA, Boudewyns A, Alexopoulos EI, Ersu R, Joosten K, et al. Obstructive sleep disordered breathing in 2- to 18-year-old children: Diagnosis and management. European Respiratory Journal. 2016;47(1):69-94. | Excluded by title |
| 175 | Kaklamanos EG, Athanasiou AE. Re: maxillary protraction appliance effect on the size of the upper airway passage. The Angle Orthod. 2008;78(5). | Excluded by title |
| 176 | Kalladka M. Dental sleep medicine. Sleep and Vigilance. 2017;1(2):137-8. | Excluded by title |
| 177 | Kamano E, Terajima M, Kitahara T, Takahashi I. Three-dimensional analysis of changes in pharyngeal airway space after mandibular setback surgery. Orthodontic Waves. 2017;76(1):1-8. | Excluded by title |
| 178 | Kanke K, Abe T, Abe M, Mori Y, Hoshi K, Takato T. In-hospital surgical treatment for haemorrhage after aesthetic mandibular osteotomy performed as an office-based day surgery: A case report. Annals of Medicine and Surgery. 2017;24:15-8. | Excluded by title |
| 179 | Kanwal B, Shetty A, Mani V, Prashanth CS, Pramod KM, Arjunan S. Esthetic Outcome and Airway Evaluation following Bi-Jaw Surgery V/S Mandibular Setback Surgery in Skeletal Class III Malocclusion Using Surgery First Approach. Ann Maxillofac Surg. 2018;8(2):270-5. | Excluded by title |
| 180 | Kapila SD, Nervina JM. CBCT in orthodontics: assessment of treatment outcomes and indications for its use. Dentomaxillofacial Radiology. 2015;44(1). | Excluded by title |
| 181 | Karabekmez FE, Keller EE, Stork JT, Regenitter FJ, Bite U. A long-term clinical and cephalometric study of cleft lip and palate patients following intraoral maxillary quadrangular le fort I osteotomy. Cleft Palate Craniofac J. 2015;52(3):311-26. | Excluded by title |
| 182 | Karloh M, Araujo CLP, Gulart AA, Reis CM, Steidle LJM, Mayer AF. The Glittre-ADL test reflects functional performance measured by physical activities of daily living in patients with chronic obstructive pulmonary disease. Braz j phys ther (Impr). 2016;20(3):223-30. | Excluded by title |
| 183 | Kasfikis G, Antoniades H, Kyrgidis A, Markovitsi E, Antoniades K. Craniofacial surgical management of a patient with systematic juvenile idiopathic arthritis and Crohn's disease. J Craniofac Surg. 2009;20(3):948-50. | Excluded by title |
| 184 | Katsuki T, Miyanoshita Y, Goto M. Long-term results of primary veloplasty for cleft palate. Japanese Journal of Plastic and Reconstructive Surgery. 1979;22(3):163-74. | Excluded by title |
| 185 | Kawakami M, Yamamoto K, Noshi T, Miyawaki S, Kirita T. Effect of surgical reduction of the tongue on dentofacial structure following mandibular setback. J Oral Maxillofac Surg. 2004;62(10):1188-92. | Excluded by title |
| 186 | Kecik D. Evaluation of Protraction Face-Mask Therapy on the Craniofacial and Upper Airway Morphology in Unilateral Cleft Lip and Palate. J Craniofac Surg. 2017;28(7):e627-e32. | Excluded by title |
| 187 | Kim J-E, Yim S, Choi J-Y, Kim S, Kim S-J, Baek S-H. Effects of the long-term use of maxillary protraction facemasks with skeletal anchorage on pharyngeal airway dimensions in growing patients with cleft lip and palate. Korean Journal of Orthodontics. 2020;50(4):238-48. | Excluded by title |
| 188 | Kim SJ, Kim YS, Park JH, Kim SW. Cephalometric predictors of therapeutic response to multilevel surgery in patients with obstructive sleep apnea. J Oral Maxillofac Surg. 2012;70(6):1404-12. | Excluded by title |
| 189 | Kim YI, Park SB, Kim JR. A study of upper airway dimensional change according to maxillary superior movement after orthognathic surgery. Korean Journal of Orthodontics. 2008;38(2):121-32. | Excluded by title |
| 190 | Kirjavainen M, Kirjavainen T. Upper airway dimensions in Class II malocclusion. Effects of headgear treatment. Angle Orthod. 2007;77(6):1046-53. | Excluded by title |
| 191 | Kitahara T, Hoshino Y, Maruyama K, In E, Takahashi I. Changes in the pharyngeal airway space and hyoid bone position after mandibular setback surgery for skeletal Class III jaw deformity in Japanese women. Am J Orthod Dentofacial Orthop. 2010;138(6):708.e1-10; discussion -9. | Excluded by title |
| 192 | Kochar GD, Chakranarayan A, Kohli S, Kohli VS, Khanna V, Jayan B, et al. Effect of surgical mandibular advancement on pharyngeal airway dimensions: a three-dimensional computed tomography study. International Journal of Oral and Maxillofacial Surgery. 2016;45(5):553-9. | Excluded by title |
| 193 | Köhler S, Wilz G, Meichsner F. Moving through predeath grief: psychological support for family caregivers of people with dementia. Dementia (14713012). 2019;18(7/8):2474-93. | Excluded by title |
| 194 | Kondo E, Ono M, Aoba TJ. Utilization of third molars in the orthodontic treatment of skeletal class III subjects with severe lateral deviation: case report. World J Orthod. 2004;5(3):201-12. | Excluded by title |
| 195 | Kreia TB. A influência do padrão respiratório no crescimento craniofacial. 2011. p. 120-. | Excluded by title |
| 196 | Kunjur J, Messiah A, Manisali M. Obstructive sleep apnoea after orthognathic surgery. International Journal of Oral and Maxillofacial Surgery. 2015;44:e250-e1. | Excluded by title |
| 197 | Kuramae M, Tavares SW, Almeida HAd, Almeida MHCd, Noüer DF. Correção da deglutição atípica associada à mordida aberta anterior: relato de caso clínico. J bras ortodon ortop facial. 2002;6(36):493-501. | Excluded by title |
| 198 | Lages PC. Impacto do uso de simbiótico na evolução de pacientes com neoplasia de vias aéreas e digestivas superiores submetidos a tratamento cirúrgico. 2016. p. 85-. | Excluded by title |
| 199 | Lan CC, Hung KF, Liao YF, Lin SW, Chen NH. Melnick-needles syndrome with obstructive sleep apnea successfully treated with nasal continuous positive airway pressure ventilation. Journal of the Formosan Medical Association. 2006;105(1):77-9. | Excluded by title |
| 200 | Langeron O, Birenbaum A, Raux M. Airway management in obese patient. Minerva Anestesiologica. 2014;80(3):382-92. | Excluded by title |
| 201 | Laraway D, Richardson D, Domingez-Gonzalez S, Duncan C. Changes in overjet and overbite following midface advancement in syndromic craniosynostosis. International Journal of Oral and Maxillofacial Surgery. 2015;44:e96. | Excluded by title |
| 202 | Laureano Filho JR, Godoy F, O'Ryan F. Orthodontic bracket lost in the airway during orthognathic surgery. Am J Orthod Dentofacial Orthop. 2008;134(2):288-90. | Excluded by title |
| 203 | Laverde-Sábogal CE, Espinosa-Almanza CJ. Fenómeno de flujo, diagnóstico diferencial de la hipoxemia refractaria en pacientes con masa mediastinal anterior. Reporte de caso. Rev colomb anestesiol. 2017;45(supl.1):66-70. | Excluded by title |
| 204 | Lee RWW, Sutherland K, Cistulli PA. Craniofacial morphology in obstructive sleep apnea: A review. Clinical Pulmonary Medicine. 2010;17(4):189-95. | Excluded by title |
| 205 | Lee UL, Oh H, Min SK, Shin JH, Kang YS, Lee WW, et al. The structural changes of upper airway and newly developed sleep breathing disorders after surgical treatment in class III malocclusion subjects. Medicine (Baltimore). 2017;96(22):e6873. | Excluded by title |
| 206 | Lepesqueur LSS. Efeitos da fibrose cística sobre o microbioma bucal e o proteoma salivar. 2019. p. 201-. | Excluded by title |
| 207 | Li L, Liu H, Cheng H, Han Y, Wang C, Chen Y, et al. CBCT evaluation of the upper airway morphological changes in growing patients of class II division 1 malocclusion with mandibular retrusion using twin block appliance: a comparative research. PLoS One. 2014;9(4):e94378. | Excluded by title |
| 208 | Li S, Wang G, Wang C, Gao X, Jin F, Yang H, et al. The REACH Trial: a Randomized Controlled Trial Assessing the Safety and Effectiveness of the Spiration® Valve System in the Treatment of Severe Emphysema. Respiration; international review of thoracic diseases. 2019;97(5):416-27. | Excluded by title |
| 209 | Li YM, Liu JL, Zhao JL, Dai J, Wang L, Chen JW. Morphological changes in the pharyngeal airway of female skeletal class III patients following bimaxillary surgery: a cone beam computed tomography evaluation. Int J Oral Maxillofac Surg. 2014;43(7):862-7. | Excluded by title |
| 210 | Linz A, Bacher M, Kagan KO, Buchenau W, Arand J, Poets CF. [Pierre Robin Sequence: interdisciplinary treatment after prenatal diagnosis]. Z Geburtshilfe Neonatol. 2011;215(3):105-8. | Excluded by title |
| 211 | Liu Y, Lowe AA, Zeng X, Fu M, Fleetham JA. Cephalometric comparisons between Chinese and Caucasian patients with obstructive sleep apnea. Am J Orthod Dentofacial Orthop. 2000;117(4):479-85. | Excluded by title |
| 212 | Liukkonen M, Vahatalo K, Peltomaki T, Tiekso J, Happonen RP. Effect of mandibular setback surgery on the posterior airway size. Int J Adult Orthodon Orthognath Surg. 2002;17(1):41-6. | Excluded by title |
| 213 | Locke SR, McKay RC, Jung ME. "I'm just too busy to exercise": reframing the negative thoughts associated with exercise-related cognitive errors. Psychology of sport and exercise. 2019;43:279-87. | Excluded by title |
| 214 | Lopes AJ, Mafort TT. Uso da técnica de Washout de Nitrogênio para avaliação do acometimento da pequena via aérea em pacientes com | Excluded by title |

| | | |
|---|---|---|
| | DPOC. Pulmão RJ. 2017;26(1):51-6. | |
| 215 | Lorenz de Sarin G, Ceccarelli AA, Rivas NH, Gastagnet SM, Bartolomé AE. Cefalograma faríngeo: 2da parte. Rev Soc Odontol La Plata. 2001;14(28):19-24. | Excluded by title |
| 216 | Luchesi KF, Kitamura S, Mourão LF. Management of dysphagia in Parkinson's disease and amyotrophic lateral sclerosis. CoDAS. 2013;25(4):358-64. | Excluded by title |
| 217 | Lukash F, Schwartz M, Korsh J, O'Brien K, Aliano K. Dynamic cleft infant maxillary orthopedics and periosteoplasty: A 25 year study. Cleft Palate-Craniofacial Journal. 2015;52(4):e126-e7. | Excluded by title |
| 218 | Ma K, Lu N, Zou F, Meng FZ. Sirtuins as novel targets in the pathogenesis of airway inflammation in bronchial asthma. Eur J Pharmacol. 2019;865:172670. | Excluded by title |
| 219 | Maas C, Poets CF. Initial treatment and early weight gain of children with Robin Sequence in Germany: a prospective epidemiological study. Arch Dis Child Fetal Neonatal Ed. 2014;99(6):F491-4. | Excluded by title |
| 220 | Machado DC, Camilo GB, Noronha AJ, Montessi OVD, Capone R, Capone D. Diagnóstico radiológico da DPOC. Pulmão RJ. 2013;22(2):45-9. | Excluded by title |
| 221 | Macri DT, Ciruffo PAD, Valdrighi HC, Vedovello SAS. Avaliação das alterações morfológicas faciais e dentárias em indivíduos adultos com incompetência labial. RGO (Porto Alegre). 2008;56(2):163-8. | Excluded by title |
| 222 | Marchetti C, Bianchi A, Merlini L, Tonelli P. Rigid internal fixation of the jaws in an adult patient with facio-scapulo-humeral muscular dystrophy: report of a case. J Craniomaxillofac Surg. 1997;25(5):275-8. | Excluded by title |
| 223 | Maricoto T, Madanelo S, Rodrigues L, Teixeira G, Valente C, Andrade L, et al. Educational interventions to improve inhaler techniques and their impact on asthma and COPD control: a pilot effectiveness-implementation trial. J bras pneumol. 2016;42(6):440-3. | Excluded by title |
| 224 | Marsan G, Cura N, Emekli U. Changes in pharyngeal (airway) morphology in Class III Turkish female patients after mandibular setback surgery. J Craniomaxillofac Surg. 2008;36(6):341-5. | Excluded by title |
| 225 | Marşan G, Vasfi Kuvat S, Öztaş E, Cura N, Süsal Z, Emekli U. Oropharyngeal airway changes following bimaxillary surgery in Class III female adults. Journal of Cranio-Maxillofacial Surgery. 2009;37(2):69-73. | Excluded by title |
| 226 | Martínez F, Pinazzo S, Moragues R, Suarez E. Complicaciones de la cirugía del plexo braquial. Neurocir - Soc Luso-Esp Neurocir. 2015;26(2):73-7. | Excluded by title |
| 227 | Martínez González C, González Barcala FJ, Belda Ramírez J, González Ros I, Alfageme Michavila I, Orejas Martínez C, et al. Recomendaciones para la evaluación médica de la capacidad laboral en el enfermo respiratorio crónico. Arch bronconeumol (Ed impr). 2013;49(11):480-90. | Excluded by title |
| 228 | Martínez LP. Macroglosia: etiología multifactorial, manejo múltiple: [revisión]. Colomb med. 2006;37(1):67-73. | Excluded by title |
| 229 | Martins AB, Tufik S, Moura SMGPT. Síndrome da apnéia-hipopnéia obstrutiva do sono. Fisiopatologia. J bras pneumol. 2007;33(1):93-100. | Excluded by title |
| 230 | Martín-Salvador A, Colodro-Amores G, Torres-Sánchez I, Moreno-Ramírez MP, Cabrera-Martos I, Valenza MC. Intervención fisioterápica durante la hospitalización en pacientes con exacerbación aguda de la enfermedad pulmonar obstructiva crónica y neumonía: un ensayo clínico aleatorizado. Med clín (Ed impr). 2016;146(7):301-4. | Excluded by title |
| 231 | Martín-Valero R, Cuesta-Vargas AI, Labajos-Manzanares M. Revisión de ensayos clínicos sobre rehabilitación respiratoria en enfermos pulmonares obstructivos crónicos. Rehabilitación (Madr, Ed impr). 2010;44(2):158-66. | Excluded by title |
| 232 | Maternal Epidural Steroids to Prevent Neonatal Exposure to Hyperthermia and Inflammation. American journal of perinatology. 2019;36(8):828-34. | Excluded by title |
| 233 | Matsumoto H, Kasai T, Hayashi H, Tabuchi H, Sekita G, Tokano T, et al. Cardiac resynchronization therapy altered the severity and pattern of sleep disordered breathing in case of heart failure with systolic dysfunction. Journal of Cardiac Failure. 2017;23(10):S53-S4. | Excluded by title |
| 234 | Matsumoto MAN, Romano FL, Ferreira JTL, Valério RA. Open bite: diagnosis, treatment and stability. Braz dent j. 2012;23(6):768-78. | Excluded by title |
| 235 | Mattos CT, Vilani GNL, Sant'Anna EF, Ruellas ACO, Maia LC. Effects of orthognathic surgery on oropharyngeal airway: A meta-analysis. International Journal of Oral and Maxillofacial Surgery. 2011;40(12):1347-56. | Excluded by title |
| 236 | Mayer G, Meier-Ewert K. Cephalometric predictors for orthopaedic mandibular advancement in obstructive sleep apnoea. Eur J Orthod. 1995;17(1):35-43. | Excluded by title |
| 237 | Mayoral Sanz P, Mayoral Herrero P. Repercusiones de la corrección quirúrgicadel prognatismo inferior en la vía aérea. Ortod esp (Ed impr). 2002;42(1):15-21. | Excluded by title |
| 238 | McKee JR. Redefining occlusion. Cranio-the Journal of Craniomandibular & Sleep Practice. 2017;35(6):343-4. | Excluded by title |
| 239 | Mendes SdL, Filgueiras VM, Ramos TB, Lacerda RHW. Associação entre padrão de crescimento facial e dimensão de orofaringe. Ortho Sci, Orthod sci pract. 2019;45(12):74-80. | Excluded by title |
| 240 | Mercuri LG. Papel de la prótesis hecha a medida a medida para la sustitución de la articulación temporomandibular. Rev esp cir oral maxilofac. 2013;35(1):1-10. | Excluded by title |
| 241 | Morales Navarro D. Atención inicial al politraumatizado maxilofacial: evaluación de la vía aérea y la circulación. Rev cuba estomatol. 2015;52(3):336-55. | Excluded by title |
| 242 | Müller-Hagedorn S, Arand J, Scholz T, Poets CF, Wiechers C. An innovative method for manufacturing the Tuebingen palatal plate for infants with Robin sequence. BMC Pediatr. 2020;20(1):103. | Excluded by title |
| 243 | Muto T, Yamazaki A, Takeda S, Sato Y. Accuracy of Predicting the Pharyngeal Airway Space on the Cephalogram After Mandibular Setback Surgery. Journal of Oral and Maxillofacial Surgery. 2008;66(6):1099-103. | Excluded by title |
| 244 | Nakagawa F, Ono T, Ishiwata Y, Kuroda T. Morphologic changes in the upper airway structure following surgical correction of mandibular prognathism. Int J Adult Orthodon Orthognath Surg. 1998;13(4):299-306. | Excluded by title |
| 245 | Nakajima Y, Nakano H, Sumida T, Yamada T, Inoue K, Sugiyama G, et al. High Le Fort I osteotomy for correction of mid-face deformity in Crouzon syndrome. Congenital Anomalies. 2016;56(5):240-2. | Excluded by title |
| 246 | Nakamura M, Yanagita T, Matsumura T, Yamashiro T, Iida S, Kamioka H. A case of severe mandibular retrognathism with bilateral condylar deformities treated with Le Fort I osteotomy and two advancement genioplasty procedures. Korean J Orthod. 2016;46(6):395-408. | Excluded by title |
| 247 | Namaki S, Maekawa N, Iwata J, Sawada K, Namaki M, Bjornland T, et al. Long-term evaluation of swallowing function before and after sagittal split ramus osteotomy. Int J Oral Maxillofac Surg. 2014;43(7):856-61. | Excluded by title |
| 248 | Ngan PW, Wei SH, Yen PK. Orthodontic treatment of the primary dentition. J Am Dent Assoc. 1988;116(3):336-40. | Excluded by title |
| 249 | Nieto Barbero MA. EPOC e hipertensión pulmonar. Arch bronconeumol (Ed impr). 2009;45(supl.4):24-30. | Excluded by title |
| 250 | Niskanen I, Kurimo J, Järnstedt J, Himanen SL, Helminen M, Peltomäki T. Effect of Maxillomandibular Advancement Surgery on Pharyngeal Airway Volume and Polysomnography Data in Obstructive Sleep Apnea Patients. J Oral Maxillofac Surg. 2019;77(8):1695-702. | Excluded by title |
| 251 | Nojima MCG. An interview with: Mark G. Hans. Dental Press Journal of Orthodontics. 2014;19(3):26-35. | Excluded by title |
| 252 | Oldfield V, Lyseng-Williamson KA. Bosentan: A review of its use in pulmonary arterial hypertension and systemic sclerosis. American Journal of Cardiovascular Drugs. 2006;6(3):189-208. | Excluded by title |
| 253 | Ortiz-Gómez JR, Palacio-Abizanda F, Fornet-Ruiz I. Failed intubation in pregnancy: Prophylaxis and management. Regional Anesthesia and Pain Medicine. 2011;36(5):E105-E13. | Excluded by title |
| 254 | Ow AT, Cheung LK. Meta-analysis of mandibular distraction osteogenesis: clinical applications and functional outcomes. Plast Reconstr Surg. 2008;121(3):54e-69e. | Excluded by title |
| 255 | Ozbek MM, Memikoglu TU, Gogen H, Lowe AA, Baspinar E. Oropharyngeal airway dimensions and functional-orthopedic treatment in skeletal Class II cases. Angle Orthod. 1998;68(4):327-36. | Excluded by title |
| 256 | Paiva JBdP. Estudo comparativo da geometria nasal e da resistência respiratória em diferentes tipos faciais. 2008. p. 174-. | Excluded by title |
| 257 | Palandi J, José P, Schmitt VM, Cechetti F, Saccani R, Bonetti LV. Análise da marcha em pacientes portadores de doença pulmonar obstrutiva crônica antes e após programa de reabilitação pulmonar. Fisioter Bras. 2017;18(4):f: 401-I:8. | Excluded by title |
| 258 | Panou E, Motro M, Ates M, Acar A, Erverdi N. Dimensional changes of maxillary sinuses and pharyngeal airway in Class III patients undergoing bimaxillary orthognathic surgery. Angle Orthod. 2013;83(5):824-31. | Excluded by title |
| 259 | Papagrigorakis MJ, Karamolegou M, Vilos G, Apostolidis C, Karamesinis K, Synodinos PN. Auriculo-condylar syndrome. Angle Orthod. 2012;82(3):556-64. | Excluded by title |

| | | |
|---|---|---|
| 260 | Papagrigorakis MJ, Karamolegou M, Vilos G, Apostolidis C, Karamesinis K, Synodinos PN. Auriculo-condylar syndrome: Diagnosis, treatment, and family history of a patient. Angle Orthodontist. 2012;82(3):556-64. | Excluded by title |
| 261 | Paramaswamy R. Anesthesia for elective bilateral sagittal slip osteotomy of the mandible and genioplasty in a young man with Klippel-Feil syndrome, Sprengel deformity, and mandibular prognathism. J Dent Anesth Pain Med. 2019;19(5):307-12. | Excluded by title |
| 262 | Pascual-Guardia S, Wodja E, Gorostiza A, López de Santamaría E, Gea J, Gáldiz JB, et al. Mejoría de la calidad de vida y capacidad de ejercicio sin cambios en la biología muscular tras entrenamiento general en pacientes con enfermedad pulmonar obstructiva crónica grave. Med clín (Ed impr). 2013;140(5):200-6. | Excluded by title |
| 263 | Pastor Vera T. Relación entre respiración oral y deglución atípica: estudio piloto de niños que presentan la característica común de lengua baja. Rev logop foniatr audiol (Ed impr). 2005;25(3):121-7. | Excluded by title |
| 264 | Pedersen C, Halvari H, Solstad BE, Bentzen M. Longitudinal trajectories of physical activity among employees participating in a worksite health promotion intervention: a latent class growth approach. Psychology of sport and exercise. 2019;43:311-20. | Excluded by title |
| 265 | Peleg AY, Choo JM, Langan KM, Edgeworth D, Keating D, Wilson J, et al. Antibiotic exposure and interpersonal variance mask the effect of ivacaftor on respiratory microbiota composition. Journal of cystic fibrosis. 2018;17(1):50-6. | Excluded by title |
| 266 | Pelegrino NRG, Lucheta PA, Sanchez FF, Faganello MM, Ferrari R, Godoy Id. Influência da massa magra corporal nas repercussões cardiopulmonares durante o teste de caminhada de seis minutos em pacientes com DPOC. J bras pneumol. 2009;35(1):20-6. | Excluded by title |
| 267 | Pereira SRA, Weckx LLM, Ortolani CLF, Bakor SF. Estudo das alterações craniofaciais e da importância da expansão rápida da maxila após adenotonsilectomia. Braz j otorhinolaryngol (Impr). 2012;78(2):111-7. | Excluded by title |
| 268 | Pinto RMdC. Caracterização clínica e inflamatória de pacientes portadores de asma grave controlada e não controlada e resposta ao acompanhamento sistemático e tratamento padronizado. 2010. p. [215]-[]. | Excluded by title |
| 269 | Pitta F, Troosters T, Probst VS, Lucas S, Decramer M, Gosselink R. Possíveis conseqüências de não se atingir a mínima atividade física diária recomendada em pacientes com doença pulmonar obstrutiva cronica estável. J bras pneumol. 2006;32(4):301-8. | Excluded by title |
| 270 | Possa SS. Tratamento com inibidor da Rho quinase em cobaias com inflamação alérgica crônica: modulação da inflamação eosinofílica, da expressão de citocinas inflamatórias, da matriz extracelular, do estresse oxidativo e da reatividade de vias aéreas. 2012. p. 159-. | Excluded by title |
| 271 | Prabhakar AR, Rai KK, Bedi S. Management of congenital bilateral temporomandibular joint ankylosis with secondary mandibular hypoplasia. J Pediatr Surg. 2008;43(10):e27-30. | Excluded by title |
| 272 | Pradel W, Lauer G, Dinger J, Eckelt U. Mandibular traction--an alternative treatment in infants with Pierre Robin sequence. J Oral Maxillofac Surg. 2009;67(10):2232-7. | Excluded by title |
| 273 | Puente-Maestu L, Villar F. Implicaciones de la calidad de vida en las decisiones quirúrgicas del cáncer de pulmón. Psicooncología (Pozuelo de Alarcón). 2006;3(2/3):393-406. | Excluded by title |
| 274 | Raboso E, Navas C, Martínez Vidal A, Vázquez R. Hamartoma fibroglandular de fosa nasal: descripción de un caso y revisión. Acta otorrinolaringol esp. 2000;51(5):445-7. | Excluded by title |
| 275 | Rachmiel A, Emodi O, Rachmiel D, Aizenbud D. Internal mandibular distraction to relieve airway obstruction in children with severe micrognathia. International Journal of Oral and Maxillofacial Surgery. 2014;43(10):1176-81. | Excluded by title |
| 276 | Radescu OD, Colosi HA, Albu S. Effects of rapid palatal expansion (RPE) and twin block mandibular advancement device (MAD) on pharyngeal structures in Class II pediatric patients from Cluj-Napoca, Romania. Cranio. 2018:1-8. | Excluded by title |
| 277 | Raskin S, Gilon Y, Limme M. [Cephalometric assessment in obstructive sleep apnea and hypopnea syndrome]. Rev Stomatol Chir Maxillofac. 2002;103(3):158-63. | Excluded by title |
| 278 | Ratjen F. Therapies from infancy: Challenges and outcomes. Pediatric Pulmonology. 2019;54:80-1. | Excluded by title |
| 279 | Raveli TB, Raveli DB, Dib LPS, Pinto PRdS. Mordida cruzada posterior e suas possibilidades de tratamento. Ortho Sci, Orthod sci pract. 2011;4(15):701-7. | Excluded by title |
| 280 | Regiane Resquetia V, Gorostiza A, Gáldiza JB, López de Santa María E, Casan Clarà P, Güell Rous R. Beneficios de un programa de rehabilitación respiratoria domiciliaria en pacientes con EPOC grave. Arch bronconeumol (Ed impr). 2007;43(11):599-604. | Excluded by title |
| 281 | Reis HV, Borghi-Silva A, Catai AM, Reis MS. Impact of CPAP on physical exercise tolerance and sympathetic-vagal balance in patients with chronic heart failure. Braz j phys ther (Impr). 2014;18(3):218-27. | Excluded by title |
| 282 | Ren YF, Isberg A, Henningsson G. The Influence of Pharyngeal Flap on Facial Growth - Long-Term Results in the Patients with Isolated Cleft-Palate. Scandinavian Journal of Plastic and Reconstructive Surgery and Hand Surgery. 1994;28(1):63-8. | Excluded by title |
| 283 | Ren YF, Isberg A, Henningsson G. The influence of pharyngeal flap on facial growth. Long term results in the patients with isolated cleft palate. Scandinavian Journal of Plastic and Reconstructive Surgery and Hand Surgery. 1994;28(1):63-8. | Excluded by title |
| 284 | Restrepo C, Santamaria A, Pelaez S, Tapias A. Oropharyngeal airway dimensions after treatment with functional appliances in class II retrognathic children. J Oral Rehabil. 2011;38(8):588-94. | Excluded by title |
| 285 | Reynders B, Vansteenkiste M, Van Puyenbroeck S, Aelterman N, De Backer M, Delrue J, et al. Coaching the coach: intervention effects on need-supportive coaching behavior and athlete motivation and engagement. Psychology of sport and exercise. 2019;43:288-300. | Excluded by title |
| 286 | Reznikov N, Dagdeviren D, Tamimi F, Glorieux F, Rauch F, Retrouvey JM. Cone-Beam Computed Tomography of Osteogenesis Imperfecta Types III and IV: Three-Dimensional Evaluation of Craniofacial Features and Upper Airways. JBMR Plus. 2019. | Excluded by title |
| 287 | Reznikov N, Dagdeviren D, Tamimi F, Glorieux F, Rauch F, Retrouvey JM. Cone-beam computed tomography of osteogenesis imperfecta types III and IV: Three-dimensional evaluation of craniofacial features and upper airways. Journal of Bone and Mineral Research. 2018;33:421. | Excluded by title |
| 288 | Ribeiro CdO, Bittencourt MAV, Brandão Filho RA. Avaliação dos efeitos da cirurgia ortognática de recuo mandibular isolado e combinado no tamanho da orofaringe. Ortho Sci, Orthod sci pract. 2011;3(13):414-7. | Excluded by title |
| 289 | Robson M, Ruddy KJ, Im S, Senkus E, Xu B, Domchek SM, et al. Patient-reported outcomes in patients with a germline BRCA mutation and HER2-negative metastatic breast cancer receiving olaparib versus chemotherapy in the OlympiAD trial. European journal of cancer. 2019;120:20-30. | Excluded by title |
| 290 | Rodrigues DB, Wolford LM, Figueiredo LMG, Adams GQ. Management of ankylosed maxillary canine with single-tooth osteotomy in conjunction with orthognathic surgery. Journal of Oral and Maxillofacial Surgery. 2014;72(12):2419.e1-.e6. | Excluded by title |
| 291 | Romanski PA, Farland LV, Tsen LC, Ginsburg ES, Lewis EI. Effect of class III and class IV obesity on oocyte retrieval complications and outcomes. Fertil Steril. 2019;111(2):294-301.e1. | Excluded by title |
| 292 | Rondeau BHM. Importance of diagnosing and treating orthodontic and orthopedic problems in children. The Functional orthodontist. 2004;21(3):passim-4, 6, 8 passim. | Excluded by title |
| 293 | Rubio-Correa I, Manzano Solo de Zaldívar D, Moreno Sánchez M, Ruíz Laza L, González Ballester D, Monje-Gil F. Melanoma mucoso en una localización extremadamente infrecuente: la base de lengua. A propósito de un caso y revisión de la literatura. Rev esp cir oral maxilofac. 2015;37(2):99-102. | Excluded by title |
| 294 | Ruiz-Laiglesia FJ, Garcés-Horna V, Formiga F. Abordaje terapéutico integral del paciente con insuficiencia cardiaca y comorbilidad. Rev clín esp (Ed impr). 2016;216(6):323-30. | Excluded by title |
| 295 | Russell SD, Lyon A, Lenihan DJ, Moreau P, Joshua D, Chng WJ, et al. Serial echocardiographic assessment of patients (PTS) with relapsed multiple myeloma (RMM) receiving carfilzomib and dexamethasone (KD) vs bortezomib and dexamethasone (VD): a substudy of the phase 3 endeavor trial (NCT01568866). Blood. 2015;126(23):4250. | Excluded by title |
| 296 | Saitoh K. Long-term changes in pharyngeal airway morphology after mandibular setback surgery. Am J Orthod Dentofacial Orthop. 2004;125(5):556-61. | Excluded by title |
| 297 | Salisbury Devicenzi JP, Grignola JC, Pascal Acuña G, Domingo E, Parma G, Trujillo Lezama P, et al. Hipertensión pulmonar en la EPOC: puesta al día. Rev urug cardiol. 2013;28(2):162-76. | Excluded by title |
| 298 | Sansone D, Flores C, Pérez C, Hanna J. Tratamiento farmacológico para el control de la crisis aguda de asma. Arch venez pueric pediatr. 2010;73(3):33-7. | Excluded by title |
| 299 | Santos LM, Ramos B, Almeida J, Loureiro CC, Cordeiro CR. The impact of weight loss beyond lung function: benefit with respect to asthma outcomes. Pulmonology. 2019;25(6):313-9. | Excluded by title |
| 300 | Sarı R, Akbaba S, Gündoğdu RH, Yazıcıoğlu M. Comparison of V-Y Flap and Limberg Flap Methods in Pilonidal Sinus Surgery. Turkish journal | Excluded by title |

| | | |
|---|---|---|
| | of colorectal disease. 2019;29(2):69-74. | |
| 301 | Schutz TC, Dominguez GC, Hallinan MP, Cunha TC, Tufik S. Class II correction improves nocturnal breathing in adolescents. Angle Orthod. 2011;81(2):222-8. | Excluded by title |
| 302 | Seeberger R, Gander E, Hoffmann J, Engel M. Surgical management of cross-bites in orthognathic surgery: Surgically assisted rapid maxillary expansion (SARME) versus two-piece maxilla. Journal of Cranio-Maxillofacial Surgery. 2015;43(7):1109-12. | Excluded by title |
| 303 | Severiche-Bueno DF, Severiche-Hernández D, Severiche-Bueno DF, Vargas MT. Síndrome de Mounier Kuhn en una paciente de 78 años con fibrosis pulmonar. Acta méd colomb. 2017;42(3):198-201. | Excluded by title |
| 304 | Shaeran TAT, Samsudin AR. Temporomandibular Joint Ankylosis Leading to Obstructive Sleep Apnea. J Craniofac Surg. 2019;30(8):e714-e7. | Excluded by title |
| 305 | Shah DH, Kim KB, McQuilling MW, Movahed R, Shah AH, Kim YI. Computational fluid dynamics for the assessment of upper airway changes in skeletal Class III patients treated with mandibular setback surgery. Angle Orthod. 2016;86(6):976-82. | Excluded by title |
| 306 | Shang H, Xue Y, Liu Y, Zhao J, He L. Modified internal mandibular distraction osteogenesis in the treatment of micrognathia secondary to temporomandibular joint ankylosis: 4-year follow-up of a case. J Craniomaxillofac Surg. 2012;40(4):373-8. | Excluded by title |
| 307 | Sidani S, Epstein DR, Fox M, Collins L. Comparing the Effects of Single- and Multiple-Component Therapies for Insomnia on Sleep Outcomes. Worldviews on evidence-based nursing / Sigma Theta Tau International, Honor Society of Nursing. 2019;16(3):195-203. | Excluded by title |
| 308 | Silva CRS, Andrade LB, Maux D, Bezerra AL, Duarte M. Effectiveness of prophylactic non-invasive ventilation on respiratory function in the postoperative phase of pediatric cardiac surgery: a randomized controlled trial. Brazilian journal of physical therapy / revista brasileira de fisioterapia. 2016;20(6):494-501. | Excluded by title |
| 309 | Silva FBNN, Cavalieri-Pereira L, Cerezetti LR, Cavalieri-Pereira S, Pedroso-Oliveira G, Rocha JLS, et al. Treatment of tmj ankylosis with alloplastic reconstruction associated with virtually planned orthognathic surgery: case report. International Journal of Oral and Maxillofacial Surgery. 2019;48:283. | Excluded by title |
| 310 | Silva KR, Marrara KT, Marino DM, Di Lorenzo VAP, Jamami M. Fraqueza muscular esquelética e intolerância ao exercício em pacientes com doença pulmonar obstrutiva crônica. Braz j phys ther (Impr). 2008;12(3):169-75. | Excluded by title |
| 311 | Silva LKd, Brasolotto AG, Berretin-Felix G. Função respiratória em indivíduos com deformidades dentofaciais. Rev CEFAC. 2015;17(3):854-63. | Excluded by title |
| 312 | Silva PRDd, Silva PPDd, Dias LPT, Domaneschi C, Uvo SAB. Tratamento cirúrgico para apnéia obstrutiva do sono. Ortodontia. 2010;43(1):63-8. | Excluded by title |
| 313 | Sing CY, Lee PHT, Leung ABK, Chung KSM, Kwok JKL, Ho RTH, et al. Managing behavioral and psychological symptoms in Chinese elderly with dementia via group-based music intervention: a cluster randomized controlled trial. Dementia (14713012). 2019;18(7/8):2785-98. | Excluded by title |
| 314 | Singla S, Utreja A, Singh SP, Lou W, Suri S. Increase in Sagittal Depth of the Bony Nasopharynx Following Maxillary Protraction in Patients With Unilateral Cleft Lip and Palate. Cleft Palate-Craniofacial Journal. 2014;51(5):585-92. | Excluded by title |
| 315 | Skilton M, Krishan A, Patel S, Sinha IP, Southern KW. Potentiators (specific therapies for class III and IV mutations) for cystic fibrosis. Cochrane Database of Systematic Reviews. 2019(1). | Excluded by title |
| 316 | Smailienė D, Intienė A, Dobradziejutė I, Kušleika G. Effect of treatment with twin-block appliances on body posture in class ii malocclusion subjects: A prospective clinical study. Medical Science Monitor. 2017;23:343-52. | Excluded by title |
| 317 | Snatos Junior JFd, Aidar LAdA, Dominguez-Rodriguez GC, Novikoff S, Abrahão M. Avaliação do espaço aéreo faríngeo em pacientes com retrognatismo mandibular tratados com aparelho Herbst. Ortodontia. 2004;37(3):8-13. | Excluded by title |
| 318 | Soares MRPS, Paula FOd, Chaves MdGAM, Assis NMdSP, Chaves Filho HDdM. Patient with Down syndrome and implant therapy: a case report. Braz dent j. 2010;21(6):550-4. | Excluded by title |
| 319 | Soni J, Shyagali TR, Bhayya DP, Shah R. Evaluation of Pharyngeal Space in Different Combinations of Class II Skeletal Malocclusion. Acta Inform Med. 2015;23(5):285-9. | Excluded by title |
| 320 | Souza R, Kairalla RA, Santos UdP, Takagaki TY, Capelozzi VL, Carvalho CRR. Diffuse panbronchiolitis: an underdiagnosed disease? Study of 4 cases in Brazil. Rev Hosp Clin Fac Med Univ São Paulo. 2002;57(4):167-74. | Excluded by title |
| 321 | Soydan SS, Bayram B, Akdeniz BS, Kayhan Z, Uckan S. Changes in difficult airway predictors following mandibular setback surgery. Int J Oral Maxillofac Surg. 2015;44(11):1351-4. | Excluded by title |
| 322 | Stanton DC. Genioplasty. Facial Plastic Surgery. 2003;19(1):75-86. | Excluded by title |
| 323 | Stelnicki EJ, Boyd JB, Nott RL, Barnavon Y, Uecker C, Henson T. Early treatment of severe mandibular hypoplasia with distraction mesenchymogenesis and bilateral free fibula flaps. J Craniofac Surg. 2001;12(4):337-48. | Excluded by title |
| 324 | Suri S, Ross RB, Tompson BD. Craniofacial morphology and adolescent facial growth in Pierre Robin sequence. American Journal of Orthodontics and Dentofacial Orthopedics. 2010;137(6):763-74. | Excluded by title |
| 325 | Tan SK, Leung WK, Tang ATH, Zwahlen RA. Effects of mandibular setback with or without maxillary advancement osteotomies on pharyngeal airways: An overview of systematic reviews. PLoS One. 2017;12(10):e0185951. | Excluded by title |
| 326 | Tangugsorn V, Krogstad O, Espeland L, Lyberg T. Obstructive sleep apnea (OSA): a cephalometric analysis of severe and non-severe OSA patients. Part I: Multiple comparison of cephalometric variables. Int J Adult Orthodon Orthognath Surg. 2000;15(2):139-52. | Excluded by title |
| 327 | Temani P, Jain P, Rathee P, Temani R. Volumetric changes in pharyngeal airway in Class II division 1 patients treated with Forsus-fixed functional appliance: A three-dimensional cone-beam computed tomography study. Contemp Clin Dent. 2016;7(1):31-5. | Excluded by title |
| 328 | Terakado K, Shiina Y, Naruse T, Sueishi K, Suzuki T, Yamaguchi H, et al. Effects of surgical orthodontic treatment on oral cavity, tongue, and pharyngeal airway: Part 1: Changes caused by mandibular setback surgery. Shikwa Gakuho. 1997;97(2):181-91. | Excluded by title |
| 329 | The Impact of Aspirin on Ultrasound Markers of Uteroplacental Flow in Low-Risk Pregnancy: secondary Analysis of a Multicenter RCT. American journal of perinatology. 2019;36(8):855-63. | Excluded by title |
| 330 | Theodore N, Aarabi B, Dhall SS, Gelb DE, Hurlbert RJ, Rozzelle CJ, et al. Transportation of patients with acute traumatic cervical spine injuries. Neurosurgery. 2013;72(SUPPL.2):35-9. | Excluded by title |
| 331 | Thofehrn C, Coutinho MSSdA, Daros CB, Assis AVd, Lima RMd, Bonin CDB, et al. Efeitos da aplicação da EPAP (Expiratory Positive Airway Pressure) sobre a tolerância ao esforço em pacientes portadores de insuficiência cardíaca. Rev bras med esporte. 2013;19(2):87-90. | Excluded by title |
| 332 | Turner MJ, Luo Y, Thomas DY, Hanrahan JW. The dual phosphodiesterase 3/4 inhibitor RPL554 stimulates rare class III and IV CFTR mutants. Am J Physiol Lung Cell Mol Physiol. 2020;318(5):L908-l20. | Excluded by title |
| 333 | Ulusoy C, Canigur Bavbek N, Tuncer BB, Tuncer C, Turkoz C, Gencturk Z. Evaluation of airway dimensions and changes in hyoid bone position following class II functional therapy with activator. Acta Odontol Scand. 2014;72(8):917-25. | Excluded by title |
| 334 | Uozumi T, Yoshikawa Y, Yokoi Y, Ando N, Taguchi A, Ogasawara T, et al. A comparative study on the morphological changes in the pharyngeal airway space before and after sagittal split ramus osteotomy. Journal of Hard Tissue Biology. 2012;21(1):35-42. | Excluded by title |
| 335 | Valero A, Navarro AM, Cuvillo Ad, Alobid I, Benito JR, Colás C, et al. Position paper on nasal obstruction: evaluation and treatment. J investig allergol clin immunol. 2018;28(2):67-90. | Excluded by title |
| 336 | Vanderveken O, Vos W, De Backer J, Wouters K, De Backer W, Dieltjens M, et al. Functional respiratory imaging to evaluate treatment effect of fixed mandibular advancement in OSA patients. Sleep. 2015;38:A195. | Excluded by title |
| 337 | Vanderveken OM, Vos W, De Backer J, Wouters K, De Backer W, Dieltjens M, et al. The role of functional respiratory imaging (FRI) in the prediction of treatment outcome with fixed mandibular advancement in OSA patients. American Journal of Respiratory and Critical Care Medicine. 2015;191. | Excluded by title |
| 338 | Vatlach S, Maas C, Poets CF. Birth prevalence and initial treatment of Robin sequence in Germany: a prospective epidemiologic study. Orphanet J Rare Dis. 2014;9:9. | Excluded by title |
| 339 | Veronezi J, Carvalho AP, Ricachinewsky C, Hoffmann A, Kobayashi DY, Piltcher OB, et al. Sleep-disordered breathing in patients with cystic fibrosis. J bras pneumol. 2015;41(4):351-7. | Excluded by title |
| 340 | Vicente González E, Adiego Leza I, Maltrana García JA, Aznar Facerías E, Ortiz García A. Fisiopatología de los trastornos respiratorios del sueño en los niños. Acta otorrinolaringol esp. 2010;61(supl.1):14-21. | Excluded by title |
| 341 | Vijay P, Pardhe N, Sunil VSB, Bajpai M, Chhibber N. Unilateral ankylosis of temporomandibular joint (TMJ) with contralateral condylar aplasia and related orthopedic deformity – syndromic or nonsyndromic? Journal of Clinical and Diagnostic Research. 2015;9(1):34-6. | Excluded by title |
| 342 | Vinereanu A, Bratu AM, Munteanu A, Teodorescu E, Tusaliu M. Craniofacial Dynamics and Treatment-Induced Functional Changes in a Case of Ectodermal Dysplasia. Romanian Journal of Oral Rehabilitation. 2018;10(1):14-9. | Excluded by title |

| | | |
|---|---|---|
| 343 | Vinoth SK, Thomas AV, Nethravathy R. Cephalomteric changes in airway dimensions with twin block therapy in growing Class II patients. Journal of Pharmacy and Bioallied Sciences. 2013;5(SUPPL.1):S25-S9. | Excluded by title |
| 344 | Vinuesa D, Ramos V, Peña A, Ruiz-Ruigómez M, Badiola J, Muñoz-Medina L, et al. Nebulized medication is not associated with nosocomial infections. A pilot study. Rev esp quimioter. 2015;28(3):154-6. | Excluded by title |
| 345 | Vos W, Vanderveken O, De Backer J, Wouters K, De Backer W, Dieltjens M, et al. Functional respiratory imaging to prediction treatment outcome of fixed mandibular advancement in OSA patients. Sleep. 2015;38:A195. | Excluded by title |
| 346 | Vos W, Vanderveken OM, De Backer J, Wouters K, De Backer W, Dieltjens M, et al. The role of functional respiratory imaging in the prediction of treatment outcome with fixed mandibular advancement in OSA patients. American Journal of Respiratory and Critical Care Medicine. 2015;191. | Excluded by title |
| 347 | Wang H, Qi S, Wang J, Cai Z, Li C. [Detection to changes in hyoid and tongue positions, and pharyngeal airway following mandibular setback surgery by cone beam CT]. Hua Xi Kou Qiang Yi Xue Za Zhi. 2012;30(6):650-4. | Excluded by title |
| 348 | Wang H, Qi S, Yan M, Zhang C, Ren S, Zhang J. [Cone-beam computed tomography evaluation of upper airway change in skeletal Class III patients after orthodontic-mandibular setback surgery]. Zhonghua Kou Qiang Yi Xue Za Zhi. 2015;50(10):615-8. | Excluded by title |
| 349 | Wang HW, Li LY, Qi SQ, Yan M. [Cone-beam computed tomography evaluation of short- and long-term airway changes in patients with skeletal Class malocclusion treated by orthodontic therapy and bimaxillary surgery with or without mandibular setback surgery]. Shanghai Kou Qiang Yi Xue. 2016;25(4):487-91. | Excluded by title |
| 350 | Wang HW, Wang JG, Qi SQ, Cai ZF, Li XH. [Three-dimensional analysis of pharyngeal airway in skeletal Class III patients after sagittal split ramus osteotomy]. Zhonghua Kou Qiang Yi Xue Za Zhi. 2012;47(4):221-4. | Excluded by title |
| 351 | Wenzel A, Williams S, Ritzau M. Relationships of changes in craniofacial morphology, head posture, and nasopharyngeal airway size following mandibular osteotomy. Am J Orthod Dentofacial Orthop. 1989;96(2):138-43. | Excluded by title |
| 352 | Werneck LC, Scola RH, Germiniani FMB, Comerlato EA, Cunha FMB. Myasthenic crisis: report of 24 cases. Arq neuropsiquiatr. 2002;60(3A):519-24. | Excluded by title |
| 353 | Wiechers C, Buchenau W, Arand J, Oertel A-F, Peters K, Mueller-Hagedorn S, et al. Mandibular growth in infants with Robin sequence treated with the Tubingen palatal plate. Head & Face Medicine. 2019;15. | Excluded by title |
| 354 | Wiechers C, Buchenau W, Arand J, Oertel AF, Peters K, Müller-Hagedorn S, et al. Mandibular growth in infants with Robin sequence treated with the Tübingen palatal plate. Head Face Med. 2019;15(1):17. | Excluded by title |
| 355 | Wolford LM, Cassano DS, Cottrell DA, El Deeb M, Karras SC, Goncalves JR. Orthognathic surgery in the young cleft patient: preliminary study on subsequent facial growth. J Oral Maxillofac Surg. 2008;66(12):2524-36. | Excluded by title |
| 356 | Xiang M, Hu B, Liu Y, Sun J, Song J. Changes in airway dimensions following functional appliances in growing patients with skeletal class II malocclusion: A systematic review and meta-analysis. Int J Pediatr Otorhinolaryngol. 2017;97:170-80. | Excluded by title |
| 357 | Xu HS, Mu XZ, Yu ZY, Feng SZ, Han JY, Zhang DS. [Experience of midfacial distraction osteogenesis in upper airway stenosis]. Zhonghua Wai Ke Za Zhi. 2008;46(8):577-80. | Excluded by title |
| 358 | Yajima Y, Oshima M, Iwai T, Kitajima H, Omura S, Tohnai I. Computational fluid dynamics study of the pharyngeal airway space before and after mandibular setback surgery in patients with mandibular prognathism. Int J Oral Maxillofac Surg. 2017;46(7):839-44. | Excluded by title |
| 359 | Yilmaz BS, Kucukkeles N. Skeletal, soft tissue, and airway changes following the alternate maxillary expansions and constrictions protocol. Angle Orthod. 2014;84(5):868-77. | Excluded by title |
| 360 | Zanelato ACT, Kochenborger R, Miranda ALRd, Bommarito S, Scanavini MA. Descrição de um método para determinação do espaço aéreo naso e bucofaríngeo. Ortho Sci, Orthod sci pract. 2009;2(7/8):672-8. | Excluded by title |
| 361 | Zhang H, Xie Y, Gu X, Li W, Zeng Y, Li S, et al. Management of Persistent Air Leaks Using Endobronchial Autologous Blood Patch and Spigot Occlusion: a Multicentre Randomized Controlled Trial in China. Respiration; international review of thoracic diseases. 2019;97(5):436-43. | Excluded by title |
| 362 | Zhang J, Zheng C, Zhu X, Zhang X, Hou Z, Zhou Z, et al. Ganji Formulation for Patients with Hepatocellular Carcinoma Who Have Undergone Surgery: a Multicenter, Randomized, Double-Blind, Controlled Trial. Evidence-based complementary & alternative medicine (ecam). 2019:1-6. | Excluded by title |
| 363 | Zheng W, Zhang X, Dong J, He J. Facial morphological characteristics of mouth breathers vs. nasal breathers: A systematic review and meta-analysis of lateral cephalometric data. Experimental and Therapeutic Medicine. 2020;19(6):3738-50. | Excluded by title |
| 364 | Ali B, Shaikh A, Fida M. CHANGES IN ORO-PHARYNGEAL AIRWAY DIMENSIONS AFTER TREATMENT WITH FUNCTIONAL APPLIANCE IN CLASS II SKELETAL PATTERN. J Ayub Med Coll Abbottabad. 2015;27(4):759-63. | Excluded by abstract |
| 365 | Ali B, Shaikh A, Fida M. Effect of Clark's twin-block appliance (CTB) and non-extraction fixed mechano-therapy on the pharyngeal dimensions of growing children. Dental Press Journal of Orthodontics. 2015;20(6):82-8. | Excluded by abstract |
| 366 | Amini M, Heravi F, Zandi B, Eslami S, Mohajerzadeh M, Rohani M. The effect of mandibular advancement device on physiologic parameters and volumetric MRI in mild to moderate obstructive sleep apnea-a randomized controlled trial. Sleep Medicine. 2017;40:e14-e5. | Excluded by abstract |
| 367 | Atik E, Gorucu-Coskuner H, Kocadereli I. Dentoskeletal and airway effects of the X-Bow appliance versus removable functional appliances (Frankel-2 and Trainer) in prepubertal Class II division 1 malocclusion patients. Australian Orthodontic Journal. 2017;33(1):3-13. | Excluded by abstract |
| 368 | Bollhalder J, Hanggi MP, Schatzle M, Markic G, Roos M, Peltomaki TA. Dentofacial and upper airway characteristics of mild and severe class II division 1 subjects. Eur J Orthod. 2013;35(4):447-53. | Excluded by abstract |
| 369 | Buschang PH, Jacob H, Carrillo R. The morphological characteristics, growth, and etiology of the hyperdivergent phenotype. Seminars in Orthodontics. 2013;19(4):212-26. | Excluded by abstract |
| 370 | Celikoglu M, Buyuk SK, Ekizer A, Unal T. Pharyngeal airway effects of Herbst and skeletal anchored Forsus FRD EZ appliances. Int J Pediatr Otorhinolaryngol. 2016;90:23-8. | Excluded by abstract |
| 371 | Chang DT, Zhou YH, Liu WT. [Evaluation of cone-beam computed tomography on upper airway changes after alternating rapid palatal expansion and constriction]. Beijing Da Xue Xue Bao Yi Xue Ban. 2017;49(4):685-90. | Excluded by abstract |
| 372 | Chang HP, Tseng YC, Chang HF. Treatment of mandibular prognathism. Journal of the Formosan Medical Association. 2006;105(10):781-90. | Excluded by abstract |
| 373 | Chokotiya H, Banthia A, Srinivasa Rao K, Choudhary K, Sharma P, Awasthi N. A study on the evaluation of pharyngeal size in different skeletal patterns: A radiographic study. Journal of Contemporary Dental Practice. 2018;19(10):1278-83. | Excluded by abstract |
| 374 | Ciavarella D, Lo Russo L, Mastrovincenzo M, Padalino S, Montaruli G, Giannatempo G, et al. Cephalometric evaluation of tongue position and airway remodelling in children treated with swallowing occlusal contact intercept appliance (S.O.C.I.A.). Int J Pediatr Otorhinolaryngol. 2014;78(11):1857-60. | Excluded by abstract |
| 375 | Da Costa MO, Monteiro P, De Silva DV, Da Rocha M, Joffly L. Sleep apnea in picnodisostosis: Case report. Sleep Science. 2020;13:59-60. | Excluded by abstract |
| 376 | de Almeida FR, Lowe AA, Otsuka R, Fastlicht S, Farbood M, Tsuiki S. Long-term sequellae of oral appliance therapy in obstructive sleep apnea patients: Part 2. Study-model analysis. American Journal of Orthodontics and Dentofacial Orthopedics. 2006;129(2):205-13. | Excluded by abstract |
| 377 | Defabjanis P. Impact of nasal airway obstruction on dentofacial development and sleep disturbances in children: preliminary notes. J Clin Pediatr Dent. 2003;27(2):95-100. | Excluded by abstract |
| 378 | Downarowicz P, Kawala B, Nowak R. Combined orthodontic and surgical treatment of skeletal class III malocclusion: A case report. Dental and Medical Problems. 2016;53(3):424-9. | Excluded by abstract |
| 379 | El H, Palomo JM. An airway study of different maxillary and mandibular sagittal positions. European Journal of Orthodontics. 2013;35(2):262-70. | Excluded by abstract |
| 380 | Fastuca R, Zecca PA, Caprioglio A. Role of mandibular displacement and airway size in improving breathing after rapid maxillary expansion. Prog Orthod. 2014;15(1):40. | Excluded by abstract |
| 381 | Fransson AMC, Tegelberg Å, Svenson BAH, Lennartsson B, Isacsson G. Influence of mandibular protruding device on airway passages and dentofacial characteristics in obstructive sleep apnea and snoring. American Journal of Orthodontics and Dentofacial Orthopedics. 2002;122(4):371-9. | Excluded by abstract |
| 382 | Fu Z, Lin Y, Ma L, Li W. Effects of maxillary protraction therapy on the pharyngeal airway in patients with repaired unilateral cleft lip and palate: A 3-dimensional computed tomographic study. Am J Orthod Dentofacial Orthop. 2016;149(5):673-82. | Excluded by abstract |
| 383 | Fujiki T, Deguchi T, Nagasaki T, Tanimoto K, Yamashiro T, Takano-Yamamoto T. Deglutitive tongue movement after correction of mandibular protrusion. Angle Orthod. 2013;83(4):591-6. | Excluded by abstract |

| | | |
|---|---|---|
| 384 | Geoghegan F, Ahrens A, McGrath C, Hagg U. An evaluation of two different mandibular advancement devices on craniofacial characteristics and upper airway dimensions of Chinese adult obstructive sleep apnea patients. Angle Orthod. 2015;85(6):962-8. | Excluded by abstract |
| 385 | Giugliano V C, Gantz V JT. Malformaciones craneofaciales y obstrucción de vía aérea superior: ¿qué y cómo corregir? Rev pediatr electrón. 2016;13(1):47-60. | Excluded by abstract |
| 386 | Gotsopoulos H, Kelly JJ, Cistulli PA. Oral appliance therapy reduces blood pressure in obstructive sleep apnea: A randomized, controlled trial. Sleep. 2004;27(5):934-41. | Excluded by abstract |
| 387 | Gregg JM, Zedalis D, Howard CW, Boyle RP, Prussin AJ. Surgical alternatives for treatment of obstructive sleep apnoea: review and case series. Ann R Australas Coll Dent Surg. 2000;15:181-4. | Excluded by abstract |
| 388 | Gupta NK, Dwivedi R, Tandan A, Singh NS. An innovative design for mandibular repositioning appliance in treating obstructive sleep apnea and snoring. Indian Journal of Public Health Research and Development. 2012;3(1):19-23. | Excluded by abstract |
| 389 | Gursoy S, Hukki J, Hurmerinta K. Five year follow-up of mandibular distraction osteogenesis on the dentofacial structures of syndromic children. Orthod Craniofac Res. 2008;11(1):57-64. | Excluded by abstract |
| 390 | Hatab N, Jezdic Z, Stefanovic N, Jelovac D, Mudrak J, Konstantinovic V. Measuring the pharyngeal airway in 3 dimensions: A preliminary study. International Journal of Oral and Maxillofacial Surgery. 2013;42(10):1260. | Excluded by abstract |
| 391 | Heidsieck DSP, Koolstra JH, de Ruiter MHT, Hoekema A, de Lange J. Biomechanical effects of a mandibular advancement device on the temporomandibular joint. Journal of Cranio-Maxillofacial Surgery. 2018;46(2):288-92. | Excluded by abstract |
| 392 | Hennig M, Schneider D, Bschorer M, Goppold K, Schön G, Bschorer R. Distraction of the mandible-a safe procedure in mild to moderate cases. Journal of Oral and Maxillofacial Surgery. 2017;75(10):e386-e7. | Excluded by abstract |
| 393 | Hong JS, Oh KM, Kim BR, Kim YJ, Park YH. Three-dimensional analysis of pharyngeal airway volume in adults with anterior position of the mandible. Am J Orthod Dentofacial Orthop. 2011;140(4):e161-9. | Excluded by abstract |
| 394 | Hourfar J, Kinzinger GSM, Feifel H, Vehr VM, Lisson JA. Effects of combined orthodontic-orthognathic treatment for class II and III correction on posterior airway space: Comparison of mono- and bignathic osteotomies. Journal of Orofacial Orthopedics. 2017;78(6):455-65. | Excluded by abstract |
| 395 | Inoue K, Hosokawa S, Sato N, Sakakibara M, Ohashi K, Sugiyama T. Relationship between malocclusion and airway volume of children in the mixed dentition period. Pediatric Pulmonology. 2019;54:S116. | Excluded by abstract |
| 396 | Jakobsone G, Neimane L, Krumina G. Two- and three-dimensional evaluation of the upper airway after bimaxillary correction of Class III malocclusion. Oral Surg Oral Med Oral Pathol Oral Radiol Endod. 2010;110(2):234-42. | Excluded by abstract |
| 397 | Keum BT, Choi SH, Choi YJ, Baik HS, Lee KJ. Effects of bodily retraction of mandibular incisors versus mandibular setback surgery on pharyngeal airway space: A comparative study. Korean Journal of Orthodontics. 2017;47(6):344-52. | Excluded by abstract |
| 398 | Kim YI, Kim SS, Son WS, Park SB. Pharyngeal airway analysis of different craniofacial morphology using cone-beam computed tomography (CBCT). Korean Journal of Orthodontics. 2009;39(3):136-45. | Excluded by abstract |
| 399 | Kim YJ, Hong JS, Hwang YI, Park YH. Three-dimensional analysis of pharyngeal airway in preadolescent children with different anteroposterior skeletal patterns. Am J Orthod Dentofacial Orthop. 2010;137(3):306.e1-11; discussion -7. | Excluded by abstract |
| 400 | Kondo E, Aoba TJ. Nonsurgical and nonextraction treatment of skeletal Class III open bite: its long-term stability. Am J Orthod Dentofacial Orthop. 2000;117(3):267-87. | Excluded by abstract |
| 401 | Learreta JA, Bono AE. Evaluación de las vías aéreas superiores en el diagnóstico ortodóncico. Ortodoncia. 1999;63(126):37-44. | Excluded by abstract |
| 402 | Li L, Wu W, Yan G, Liu L, Liu H, Li G, et al. Analogue simulation of pharyngeal airflow response to Twin Block treatment in growing patients with Class II(1) and mandibular retrognathia. Sci Rep. 2016;6:26012. | Excluded by abstract |
| 403 | Li ZM, Wu J, Men HY, Li HF. [Cone-beam CT study for the oropharyngeal airway volume and hyoid position of adults Class III skeletal malocclusion]. Shanghai Kou Qiang Yi Xue. 2015;24(3):351-5. | Excluded by abstract |
| 404 | Licciardello V, Carnemolla ME. Positional changes of hyoid bone and cranio-cervical posture following class III orthopedic treatment. Minerva Stomatol. 2005;54(11-12):635-46. | Excluded by abstract |
| 405 | Marino A, Ranieri R, Chiarotti F, Villa MP, Malagola C. Rapid maxillary expansion in children with Obstructive Sleep Apnoea Syndrome (OSAS). Eur J Paediatr Dent. 2012;13(1):57-63. | Excluded by abstract |
| 406 | McDonald JP. Airway problems in children: Can the orthodontist help? Annals Academy of Medicine Singapore. 1995;24(1):158-62. | Excluded by abstract |
| 407 | Mocellin M, Fugmann EA, Gavazzoni FB, Ataide ALd, Ouriques FL, Herrero Júnior F. Estudo cefalométrico-radiográfico e otorrinolaringológico correlacionando o grau de obstrução nasal e o padrão de crescimento facial em pacientes não tratados ortodonticamente. Rev bras otorrinolaringol. 2000;66(2):116-20. | Excluded by abstract |
| 408 | Molina F, Ortiz Monasterio F, de la Paz Aguilar M, Barrera J. Maxillary distraction: aesthetic and functional benefits in cleft lip-palate and prognathic patients during mixed dentition. Plast Reconstr Surg. 1998;101(4):951-63. | Excluded by abstract |
| 409 | Morales-Fernandez M, Iglesias-Linares A, Yanez-Vico RM, Mendoza-Mendoza A, Solano-Reina E. Bone- and dentoalveolar-anchored dentofacial orthopedics for Class III malocclusion: New approaches, similar objectives? A systematic review. Angle Orthodontist. 2013;83(3):540-52. | Excluded by abstract |
| 410 | Mucedero M, Pavoni C, Cozza P. Skeletal components and classification of Class III malocclusions. Mondo Ortodontico. 2009;34(3):165-78. | Excluded by abstract |
| 411 | Nguyen TV, Loudon ME. Upper airway obstruction and resultant growth factors influencing malocclusions. Int J Orthod Milwaukee. 2015;26(1):43-6. | Excluded by abstract |
| 412 | Ottaviano G, Maculan P, Borghetto G, Favero V, Galletti B, Savietto E, et al. Nasal function before and after rapid maxillary expansion in children: A randomized, prospective, controlled study. International Journal of Pediatric Otorhinolaryngology. 2018;115:133-8. | Excluded by abstract |
| 413 | Padma A, Ramakrishnan N, Narayanan V. Management of obstructive sleep apnea: A dental perspective. Indian J Dent Res. 2007;18(4):201-9. | Excluded by abstract |
| 414 | Paolucci ECd. Asociación entre mofología craneocervical y anomalías ortodóncicas con obstrucción respiratoria. Rev Fac Odontol [Córdoba]. 1992;19/20(1/2):79-85. | Excluded by abstract |
| 415 | Pirila-Parkkinen K, Pirttiniemi P, Nieminen P, Lopponen H, Tolonen U, Uotila R, et al. Cervical headgear therapy as a factor in obstructive sleep apnea syndrome. Pediatr Dent. 1999;21(1):39-45. | Excluded by abstract |
| 416 | Poets CF, Bacher M. Treatment of upper airway obstruction and feeding problems in Robin-like phenotype. Journal of Pediatrics. 2011;159(6):887-92. | Excluded by abstract |
| 417 | Prabhat KC, Goyal L, Bey A, Maheshwari S. Recent advances in the management of obstructive sleep apnea: The dental perspective. J Nat Sci Biol Med. 2012;3(2):113-7. | Excluded by abstract |
| 418 | Rahimi H. Re: Effects of maxillary protraction and fixed appliance therapy on the pharyngeal airway. Angle Orthod. 2009;79(5):i; author reply ii. | Excluded by abstract |
| 419 | Raskin S, Limme M. [Obstructive sleep apnea syndrome: the orthodontist's viewpoint]. Rev Belge Med Dent (1984). 1991;46(4):33-7. | Excluded by abstract |
| 420 | Rose E, Schessl J. Orthodontic procedures in the treatment of obstructive sleep apnea in children. J Orofac Orthop. 2006;67(1):58-67. | Excluded by abstract |
| 421 | Schendel S, Powell N, Jacobson R. Maxillary, mandibular, and chin advancement: treatment planning based on airway anatomy in obstructive sleep apnea. J Oral Maxillofac Surg. 2011;69(3):663-76. | Excluded by abstract |
| 422 | Shibata M, Tanikawa C, Yashiro K, Shintaku Y, Kogo M, Yamashiro T. Early dentofacial orthopedic treatment of a patient with maxillary hypoplasia and congenital central hypoventilation syndrome. Orthodontic Waves. 2014;73(1):29-33. | Excluded by abstract |
| 423 | Smahel Z, Mullerova Z, Nejedly A, Horak I. Changes in craniofacial development due to modifications of the treatment of unilateral cleft lip and palate. Cleft Palate Craniofac J. 1998;35(3):240-7. | Excluded by abstract |
| 424 | Sokucu O, Doruk C, Uysal OI. Comparison of the effects of RME and fan-type RME on nasal airway by using acoustic rhinometry. Angle Orthod. 2010;80(5):870-5. | Excluded by abstract |
| 425 | Solano-Mendoza B, Iglesias-Linares A, Yanez-Vico RM, Mendoza-Mendoza A, Alio-Sanz JJ, Solano-Reina E. Maxillary protraction at early ages. | Excluded by |

| | | |
|---|---|---|
| | The revolution of new bone anchorage appliances. J Clin Pediatr Dent. 2012;37(2):219-29. | abstract |
| 426 | Solow B, Tallgren A. Head Posture and Craniofacial Morphology. American Journal of Physical Anthropology. 1976;44(3):417-35. | Excluded by abstract |
| 427 | Stellzig-Eisenhauer A, Meyer-Marcotty P. [Interaction between otorhinolaryngology and orthodontics: correlation between the nasopharyngeal airway and the craniofacial complex]. Laryngorhinootologie. 2010;89 Suppl 1:S72-8. | Excluded by abstract |
| 428 | Sutherland K, Chan ASL, Cistulli PA. Three-dimensional assessment of anatomical balance and oral appliance treatment outcome in obstructive sleep apnoea. Sleep and breathing. 2016;20(3):903-10. | Excluded by abstract |
| 429 | Tangugsorn V, Skatvedt O, Krogstad O, Lyberg T. Obstructive sleep apnoea: a cephalometric study. Part I. Cervico-craniofacial skeletal morphology. Eur J Orthod. 1995;17(1):45-56. | Excluded by abstract |
| 430 | Tapia IE, Marcus CL. Newer treatment modalities for pediatric obstructive sleep apnea. Paediatric Respiratory Reviews. 2013;14(3):199-203. | Excluded by abstract |
| 431 | Tarkar JS, Parashar S, Gupta G, Bhardwaj P, Maurya RK, Singh A, et al. An Evaluation of Upper and Lower Pharyngeal Airway Width, Tongue Posture and Hyoid Bone Position in Subjects with Different Growth Patterns. J Clin Diagn Res. 2016;10(1):Zc79-83. | Excluded by abstract |
| 432 | Twardokęs M, Paluch Z, Stelmańska K, Ura-Sabat K, Gamrot-Wrzoł M, Zieliński M, et al. Morphometric relationships between the upper respiratory tract and the craniofacial bone structures. Journal of Stomatology. 2016;69(5):482-91. | Excluded by abstract |
| 433 | Ulkur F, Uyar VO, Ozdemir F, Guzel MZ. Interdisciplinary orthognathic treatment of high angle class III malocclusion. Niger J Clin Pract. 2015;18(1):144-8. | Excluded by abstract |
| 434 | Wang W, Di C, Mona S, Wang L, Hans M. Tongue function: An underrecognized component in the treatment of obstructive sleep apnea with mandibular repositioning appliance. Canadian Respiratory Journal. 2018;2018. | Excluded by abstract |
| 435 | Yassaei S, Bahrololoomi Z, Sorush M. Changes of tongue position and oropharynx following treatment with functional appliance. J Clin Pediatr Dent. 2007;31(4):287-90. | Excluded by abstract |
| 436 | Miotti A, Miotti F, Miotti B. [Teleradiographic study of the changes of the pharyngeal and of the intermaxillary space in cases of orthodontically treated class III malocclusion]. Mondo ortodontico. 1979;4(2):25-7. | Excluded; missing full-text |
| 437 | Miotti A, Miotti F, Miotti B. [Dento-alveolar skeletal and pharyngeal space modifications in cases of Class III, treated orthodontically]. Mondo ortodontico. 1979;4(2):53-69. | Excluded; missing full-text |
| 438 | Büyükçavuş MH. Alternate Rapid Maxillary Expansion and Constriction (Alt-RAMEC) protocol: A comprehensive literature review. Turkish Journal of Orthodontics. 2019;32(1):47-51. | Excluded; no clinical study |
| 439 | Indriksone I, Jakobsone G. The upper airway dimensions in different sagittal craniofacial patterns: a systematic review. Stomatologija. 2014;16(3):109-17. | Excluded; no clinical study |
| 440 | Kachinski A, Azanha JM, Campos PD, Corrêa CdC, Campos LD. Avaliação das vias aéreas superiores por meio de tomografia computadorizada de feixe cônico em indivíduos submetidos à cirurgia ortognática: revisão de literatura. Rev Salusvita (Online). 2018;37(2):405-19. | Excluded; no clinical study |
| 441 | Oliveira DMCNSd, Santana VC, Santos Júnior JHd. Avaliação do espaço aéreo faríngeo em pacientes submetidos ao tratamento ortopédico-ortodôntico – revisão de literatura. Ortho Sci, Orthod sci pract. 2014;7(26):230-7. | Excluded; no clinical study |
| 442 | Lee WC, Tu YK, Huang CS, Chen R, Fu MW, Fu E. Pharyngeal airway changes following maxillary expansion or protraction: A meta-analysis. Orthodontics & Craniofacial Research. 2018;21(1):4-11. | Excluded; review |
| 443 | Ming Y, Hu Y, Li Y, Yu J, He H, Zheng L. Effects of maxillary protraction appliances on airway dimensions in growing class III maxillary retrognathic patients: A systematic review and meta-analysis. International Journal of Pediatric Otorhinolaryngology. 2018;105:138-45. | Excluded; review |
| 444 | Mousoulea S, Tsolakis I, Ferdianakis E, Tsolakis AI. The Effect of Chin-cup Therapy in Class III Malocclusion: A Systematic Review. Open Dentistry Journal. 2016;10:664-79. | Excluded; review |
| 445 | Borborema dos Santos VD, Assis GMd, Pereira da Silva JS, Rocha Germano A. Glosectomía parcial en paciente portador del síndrome de Beckwith-Wiedemann: relato del caso presentation of a case. Revista Española de Cirugía Oral y Maxilofacial. 2015;37(4):202-6. | Excluded; case report / series |
| 446 | Hoshijima M, Honjo T, Moritani N, Iida S, Yamashiro T, Kamioka H. Maxillary Advancement for Unilateral Crossbite in a Patient with Sleep Apnea Syndrome. Acta Medica Okayama. 2015;69(3):177-82. | Excluded; case report / series |
| 447 | Guimarães MdLR, Pereira JBB, Jardim FFT, Costa TMMd, Becattini PR, Hermont AP. Efetividade em Longo-Prazo de dois Aparelhos Intraorais no Tratamento da Apneia Obstrutiva do Sono: Relato de um Caso. UNOPAR Cient, Ciênc biol saude. 2014;16(4). | Excluded; case report / series |
| 448 | Afzal E, Fida M. Evaluation of the change in the tongue posture and in the hyoid bone position after Twin Block appliance therapy in skeletal class II subjects. Dental and Medical Problems. 2019;56(4):379-84. | Excluded; no Class III malocclusion |
| 449 | Alhammadi MS, Elfeky HY, Fayed MS, Ishaq RAR, Halboub E, Al-Mashraqi AA. Three-dimensional skeletal and pharyngeal airway changes following therapy with functional appliances in growing skeletal Class II malocclusion patients : A controlled clinical trial. J Orofac Orthop. 2019;80(5):254-65. | Excluded; no Class III malocclusion |
| 450 | Amuk NG, Kurt G, Baysal A, Turker G. Changes in pharyngeal airway dimensions following incremental and maximum bite advancement during Herbst-rapid palatal expander appliance therapy in late adolescent and young adult patients: a randomized non-controlled prospective clinical study. European Journal of Orthodontics. 2019;41(3):322-30. | Excluded; no Class III malocclusion |
| 451 | An S-Y, Park S-Y, Youn-Soo S, 홍기상, 김아현. Oropharyngeal Airway Dimensional Changes after Treatment with Myobrace (Trainer for Kids (T4K)) in Class II Retrognathic Children. Journal of Korean academy of Advanced General Dentistry. 2015;4(2):39-50. | Excluded; no Class III malocclusion |
| 452 | Anusuya V, Jena AK, Sharan J. Effects of functional appliance treatment on pharyngeal airway passage dimensions in Class II malocclusion subjects with retrognathic mandibles: A systematic review. Apos Trends in Orthodontics. 2019;9(3):138-48. | Excluded; no Class III malocclusion |
| 453 | Basciftci FA, Mutlu N, Karaman AI, Malkoc S, Kucukkolbasi H. Does the timing and method of rapid maxillary expansion have an effect on the changes in nasal dimensions? Angle Orthod. 2002;72(2):118-23. | Excluded; no Class III malocclusion |
| 454 | Chiari S, Romsdorfer P, Swoboda H, Bantleon HP, Freudenthaler J. Effects of rapid maxillary expansion on the airways and ears - A pilot study. European Journal of Orthodontics. 2009;31(2):135-41. | Excluded; no Class III malocclusion |
| 455 | Chiari S, Romsdorfer P, Swoboda H, Bantleon H-P, Freudenthaler J. Effects of rapid maxillary expansion on the airways and ears-025EFa pilot study. European Journal of Orthodontics. 2009;31(2):135-41. | Excluded; no Class III malocclusion |
| 456 | Chiari S, Romsdorfer P, Swoboda H, Bantleon HP, Freudenthaler J. Effects of rapid maxillary expansion on the airways and ears--a pilot study. Eur J Orthod. 2009;31(2):135-41. | Excluded; no Class III malocclusion |
| 457 | Coceancig P. Introducing the IMDO protocol into practical private orthognathic practice for first line management of adolescent class ii malocclusion. International Journal of Oral and Maxillofacial Surgery. 2019;48:114. | Excluded; no Class III malocclusion |
| 458 | Cortese M, Pigato G, Casiraghi G, Ferrari M, Bianco E, Maddalone M. Evaluation of the oropharyngeal airway space in class II malocclusion treated with mandibular activator: A retrospective study. Journal of Contemporary Dental Practice. 2020;21(6):666-72. | Excluded; no Class III malocclusion |
| 459 | Deng L, He H, Ngan P. 2-D and 3-D Evaluation of Upper Airway Changes After Maxillary Expansion with the Alt-RAMEC Protocol. Wuhan Daxue Xuebao (Yixue Ban). 2016;37(6):960-4. | Excluded; no Class III malocclusion |
| 460 | Durán-Cantolla J, Aizpuru F, Miranda E, Alkhraisat M-H, Anitua E, Crovetto-Martínez R, et al. Efficacy of mandibular advancement device in the treatment of obstructive sleep apnea syndrome: A randomized controlled crossover clinical trial. Med oral patol oral cir bucal (Internet). 2015;20(5):605-15. | Excluded; no Class III malocclusion |
| 461 | Jacob HB, Santo MD, Bosio JA. Extrações ortodônticas de pré-molares e seus efeitos nas vias aéreas respiratórias superiores revisão da | Excluded; no |

| | | |
|---|---|---|
| | literatura. Ortho Sci, Orthod sci pract. 2017;10(37):106-11. | Class III malocclusion |
| 462 | Kirjavainen M, Kirjavainen T. Upper airway dimensions in class II Malocclusion - Effects of headgear treatment. Angle Orthodontist. 2007;77(6):1046-53. | Excluded; no Class III malocclusion |
| 463 | Machado-Júnior A-J, Signorelli L-G, Zancanella E, Crespo A-N. Randomized controlled study of a mandibular advancement appliance for the treatment of obstructive sleep apnea in children: A pilot study. Med oral patol oral cir bucal (Internet). 2016;21(4):e403-e7. | Excluded; no Class III malocclusion |
| 464 | Prescinotto R, Haddad FLM, Fukuchi I, Gregório LC, Cunali PA, Tufik S, et al. Impact of upper airway abnormalities on the success and adherence to mandibular advancement device treatment in patients with Obstructive Sleep Apnea Syndrome. Braz j otorhinolaryngol (Impr). 2015;81(6):663-70. | Excluded; no Class III malocclusion |
| 465 | Rădescu OD, Colosi HA, Albu S. Effects of rapid palatal expansion (RPE) and twin block mandibular advancement device (MAD) on pharyngeal structures in Class II pediatric patients from Cluj-Napoca, Romania. Cranio. 2020;38(1):22-9. | Excluded; no Class III malocclusion |
| 466 | Silva FBNN, Cavalieri-Pereira L, Pedroso-Oliveira G, Rocha JLS, Brancher GQB. Orthognathic surgery with mandibular osteotomy modified for OSAS treatment in class II patient. International Journal of Oral and Maxillofacial Surgery. 2019;48:277. | Excluded; no Class III malocclusion |
| 467 | Silva Filho OGd, Lara TS, Ayub PV, Ohashi ASC, Bertoz FA. Photographic assessment of nasal morphology following rapid maxillary expansion in children. J appl oral sci. 2011;19(5):535-43. | Excluded; no Class III malocclusion |
| 468 | Singh GD, Garcia-Motta AV, Hang WM. Evaluation of the posterior airway space following Biobloc therapy: geometric morphometrics. Cranio. 2007;25(2):84-9. | Excluded; no Class III malocclusion |
| 469 | Sökücü O, Doruk C, Uysal OI. Comparison of the effects of RME and fan-type RME on nasal airway by using acoustic rhinometry Oral. Angle Orthodontist. 2010;80(5):870-5. | Excluded; no Class III malocclusion |
| 470 | Teixeira AOdB. Uso de aparelho de protrusão mandibular como recurso para tratamento da síndrome da apnéia obstrutiva do sono. 2008. p. 119-. | Excluded; no Class III malocclusion |
| 471 | Thakur VK, Londhe SM, Kumar P, Sharma M, Jain A, Pradhan I. Evaluation and quantification of airway changes in Class II division 1 patients undergoing myofunctional therapy using twin block appliance. Medical Journal Armed Forces India. 2020. | Excluded; no Class III malocclusion |
| 472 | Wiedemeyer V, Berger M, Martini M, Kramer FJ, Heim N. Predictability of pharyngeal airway space dimension changes after orthognathic surgery in class II patients: A mathematical approach. Journal of Cranio-Maxillofacial Surgery. 2019;47(10):1504-9. | Excluded; no Class III malocclusion |
| 473 | Cunha TCA, GuimarÃEs TdM, Schultz TCB, Almeida FRd, Cunha TM, Simamoto Junior PC, et al. Predictors of success for mandibular repositioning appliance in obstructive sleep apnea syndrome. Braz oral res (Online). 2017;31:e37-e. | Excluded; obstructive sleep apnea patients |
| 474 | Freire-Maia BAV. A influência da obstrução das vias aéreas superiores na determinação do tipo facial. 2009. p. 96-. | Excluded; obstructive sleep apnea patients |
| 475 | Juliano ML, Machado MAC, Carvalho LBCd, Prado LBFd, Prado GFd. Mouth breathing children have cephalometric patterns similar to those of adult patients with obstructive sleep apnea syndrome. Arq neuropsiquiatr. 2009;67(3b):860-5. | Excluded; obstructive sleep apnea patients |
| 476 | Li Y, Wu J, Guo J, Yu L, Wang J, Li X, et al. The efficacy of different treatment approaches for pediatric OSAHS patients with mandibular retrognathia: study protocol for a multicenter randomized controlled trial. Trials. 2020;21(1):595. | Excluded; obstructive sleep apnea patients |
| 477 | Mayer G, Meierewert K. Cephalometric Predictors for Orthopedic Mandibular Advancement in Obstructive Sleep-Apnea. European Journal of Orthodontics. 1995;17(1):35-43. | Excluded; obstructive sleep apnea patients |
| 478 | Pliska BT, Nam H, Chen H, Lowe AA, Almeida FR. Obstructive Sleep Apnea and Mandibular Advancement Splints: Occlusal Effects and Progression of Changes Associated with a Decade of Treatment. Journal of Clinical Sleep Medicine. 2014;10(12):1285-91. | Excluded; obstructive sleep apnea patients |
| 479 | Quintela MdM, Lima Júnior A, Sallum RA, Pacheco Filho F, Flório FM, Motta RHL. Avaliação da eficácia de um aparelho de avanço mandibular semiflexível na apneia obstrutiva do sono: estudo clínico e polissonográfico. Ortodontia. 2013;46(4):358-69. | Excluded; obstructive sleep apnea patients |
| 480 | Quo S, Lo LF, Guilleminault C. Maxillary protraction to treat pediatric obstructive sleep apnea and maxillary retrusion: a preliminary report. Sleep Med. 2019;60:60-8. | Excluded; obstructive sleep apnea patients |
| 481 | Quo SD, Hyunh N, Guilleminault C. Bimaxillary expansion therapy for pediatric sleep-disordered breathing. Sleep Medicine. 2017;30:45-51. | Excluded; obstructive sleep apnea patients |
| 482 | Schendel S, Powell N, Jacobson R. Maxillary, Mandibular, and Chin Advancement: Treatment Planning Based on, Airway Anatomy in Obstructive Sheep Apnea. Journal of Oral and Maxillofacial Surgery. 2011;69(3):663-76. | Excluded; obstructive sleep apnea patients |
| 483 | Segovia D. Síndrome de apnea-hipoapnea obstructiva del sueño en adultos. Detección y tratamiento en ortodoncia. Ortodoncia. 2016;80(160):42-8. | Excluded; obstructive sleep apnea patients |
| 484 | Smatt Y, Ferri J. Retrospective study of 18 patients treated by maxillomandibular advancement with adjunctive procedures for obstructive sleep apnea syndrome. Journal of Craniofacial Surgery. 2005;16(5):770-7. | Excluded; obstructive sleep apnea patients |
| 485 | Sturzinger GPS, Lima BC, Rodrigues GGN, Ferreira VF, Oliveira SM, Vianna ALB, et al. Hilotherapy treatment in postoperative surgery in a patient with severe osahs. International Journal of Oral and Maxillofacial Surgery. 2019;48:165. | Excluded; obstructive sleep apnea patients |
| 486 | Vinha PP, Santos GP, Bandão G, Fagnani Filho A. Ronco e apneia do sono: apresentação de novo dispositivo intra-oral e protocolo de tratamento. RGO (Porto Alegre). 2010;58(4):515-20. | Excluded; obstructive sleep apnea patients |
| 487 | Abramova MY, Alimova AV, Slabkovskaya AB, Alekseeva AA. Analysis of oral soft tissues, airways and tongue by the results of complex rehabilitation of patients with angle class III malocclusion. Annals of Tropical Medicine and Public Health. 2018;9(Special Issue):S611. | Excluded; no orthopedic treatment |
| 488 | Alves PVM, Zhao L, O'Gara M, Patel PK, Bolognese AM. Three-Dimensional Cephalometric Study of Upper Airway Space in Skeletal Class II and III Healthy Patients. Journal of Craniofacial Surgery. 2008;19(6):1497-507. | Excluded; no orthopedic treatment |
| 489 | Arkuszewski P, Gaszynska E, Przygonski A. A method for determination of tongue size in patients with mandibular prognathism. Roczniki Pomorskiej Akademii Medycznej w Szczecinie. 2006;52(Suppl. 3):125-9. | Excluded; no orthopedic |

| | | |
|---|---|---|
| | | treatment |
| 490 | Athanasiou AE, Toutountzakis N, Mavreas D, Ritzau M, Wenzel A. Alterations of Hyoid Bone Position and Pharyngeal Depth and Their Relationship after Surgical-Correction of Mandibular Prognathism. American Journal of Orthodontics and Dentofacial Orthopedics. 1991;100(3):259-65. | Excluded; no orthopedic treatment |
| 491 | Azevedo WRdS, Feitoza CC, Vargas Junior CS, Pizzol KEDC. Changes in head posture, hyoid bone position and airway dimensions after orthognatic surgery. Braz j oral sci. 2018;17:e18030-e. | Excluded; no orthopedic treatment |
| 492 | Chou PY, Denadai R, Yao CF, Chen YA, Chang CS, Lin CC, et al. History and Evolution of Orthognathic Surgery at Chang Gung Craniofacial Center: Lessons Learned From 35-Year Experience. Ann Plast Surg. 2020;84(1S Suppl 1):S60-s8. | Excluded; no orthopedic treatment |
| 493 | Coeugniet E, Pellerin P, Wolber A, Dhellemmes P, Vinchon M. Fifteen years of experience with the midfacial distraction without maxillary osteotomy protocol. Childs Nervous System. 2014;30(4):681-8. | Excluded; no orthopedic treatment |
| 494 | Cota R, Goto A. Surgical treatment for dentofacial deformities: class III patient with mandibular laterodeviation and anterior open bite. International Journal of Oral and Maxillofacial Surgery. 2019;48:191. | Excluded; no orthopedic treatment |
| 495 | da Silva Machado V, Cantharino de Carvalho BA, Vedovello SAS, Valdrighi H, Santamaria Júnior M. Pharyngeal Airway Modifications in Skeletal Class III Patients Undergoing Bimaxillary Advancement Surgery. J Oral Maxillofac Surg. 2019;77(10):2126.e1-.e8. | Excluded; no orthopedic treatment |
| 496 | Gornic C, Nascimento PPd, Melgaço CA, Ruellas ACdO, Medeiros PJDA, Sant'Anna EF. Análise cefalométrica das vias aéreas superiores de pacientes Classe III submetidos a tratamento ortocirúrgico subjected to orthosurgical treatment. Dental Press Journal of Orthodontics. 2011;16(5):82-8. | Excluded; no orthopedic treatment |
| 497 | Hochban W, Schurmann R, Brandenburg U, Conradt R. Mandibular setback for surgical correction of mandibular hyperplasia - Does it provoke sleep related breathing disorders? International Journal of Oral and Maxillofacial Surgery. 1996;25(5):333-8. | Excluded; no orthopedic treatment |
| 498 | Hourfar J, Kinzinger GSM, Feifel H, Vehr VM, Lisson JA. Effects of combined orthodontic-orthognathic treatment for class II and III correction on posterior airway space. Journal of Orofacial Orthopedics-Fortschritte Der Kieferorthopadie. 2017;78(6):455-65. | Excluded; no orthopedic treatment |
| 499 | Jayaratne YSN, Zwahlen RA. The Oropharyngeal Airway in Young Adults with Skeletal Class II and Class III Deformities: A 3-D Morphometric Analysis. Plos One. 2016;11(2). | Excluded; no orthopedic treatment |
| 500 | Lee SH, 김정재. Changes of airway after orthognathic surgery for patients with skeletal class III malocclusion. Journal of Korean society of Dental Hygiene. 2018;18(4):525-33. | Excluded; no orthopedic treatment |
| 501 | Lye KW. Effect of orthognathic surgery on the posterior airway space (PAS). Annals Academy of Medicine Singapore. 2008;37(8):677-82. | Excluded; no orthopedic treatment |
| 502 | Moscarino S, Kötter F, Brandt M, Modabber A, Kniha K, Hölzle F, et al. Influence of different surgical concepts for moderate skeletal class II and III treatment on the nasopharyngeal airway space. Journal of Cranio-Maxillofacial Surgery. 2019;47(10):1489-97. | Excluded; no orthopedic treatment |
| 503 | On SW, Kim HJ, Cho DH, Moon YR, Song SI. Silent Changes in Sleep Quality Following Mandibular Setback Surgery in Patients with Skeletal Class III Malocclusion: A Prospective Study. Scientific Reports. 2019;9. | Excluded; no orthopedic treatment |
| 504 | Parsi GK, Alsulaiman AA, Kotak B, Mehra P, Will LA, Motro M. Volumetric changes of the upper airway following maxillary and mandibular advancement using cone beam computed tomography. International Journal of Oral and Maxillofacial Surgery. 2019;48(2):203-10. | Excluded; no orthopedic treatment |
| 505 | Rabelo Bozzini MF, Valladares-Neto J, de Paiva JB, Rino-Neto J. Sex differences in pharyngeal airway morphology in adults with skeletal Class III malocclusion. Cranio-the Journal of Craniomandibular & Sleep Practice. 2018;36(2):98-105. | Excluded; no orthopedic treatment |
| 506 | Saitoh K. Long-term changes in pharyngeal airway morphology after mandibular set-back surgery. American Journal of Orthodontics and Dentofacial Orthopedics. 2004;125(5):556-61. | Excluded; no orthopedic treatment |
| 507 | Tseng Y-C, Hsiao S-Y, Cheng J-H, Hsu K-J, Chen C-M. Postoperative Skeletal Stability and Pharyngeal Airway: Counterclockwise versus Clockwise Rotation during Mandibular Setback Surgery. Biomed Research International. 2020;2020. | Excluded; no orthopedic treatment |
| 508 | Tseng Y-C, Lai S, Lee H-E, Chen K-K, Chen C-M. Are Hyoid Bone and Tongue the Risk Factors Contributing to Postoperative Relapse for Mandibular Prognathism? Biomed Research International. 2016;2016. | Excluded; no orthopedic treatment |
| 509 | Wang H-W, Li L-Y, Qi S-Q, Yan M. [Cone-beam computed tomography evaluation of short- and long-term airway changes in patients with skeletal Class Ⅲ malocclusion treated by orthodontic therapy and bimaxillary surgery with or without mandibular setback surgery]. Shanghai kou qiang yi xue = Shanghai journal of stomatology. 2016;25(4):487-91. | Excluded; no orthopedic treatment |
| 510 | Wenzel A, Williams S, Ritzau M. Changes in Head Posture and Nasopharyngeal Airway Following Surgical-Correction of Mandibular Prognathism. European Journal of Orthodontics. 1989;11(1):37-42. | Excluded; no orthopedic treatment |
| 511 | Yoshida K, Sakuda M, Takada K, Tsuchiya M. Changes of Tongue Function after Orthognathic Surgery in Class-Iii Cases. Journal of Dental Research. 1987;66:161-. | Excluded; no orthopedic treatment |
| 512 | 이규홍, 황용인, 김윤지, 천세환, 김형욱, 박준우, et al. Changes in tongue position, airway width, gonial angle, lower facial height after bilateral sagittal split ramus osteotomy in mandibular prognathic patients. Journal of the Korean Association of Oral and Maxillofacial Surgeons. 2007;33(2):109-13. | Excluded; no orthopedic treatment |
| 513 | Chen X, Liu D, Liu J, Wu Z, Xie Y, Li L, et al. Three-Dimensional Evaluation of the Upper Airway Morphological Changes in Growing Patients with Skeletal Class III Malocclusion Treated by Protraction Headgear and Rapid Palatal Expansion: A Comparative Research. PLoS One. 2015;10(8):e0135273. | Excluded; no untreated Class III group |
| 514 | Danaie SM, Salehi P. Cephalometric evaluation of class-III patients with chin cap and tongue guard. J Indian Soc Pedod Prev Dent. 2005;23(2):63-6. | Excluded; no untreated Class III group |
| 515 | Fareen N, Alam MK, Khamis MF, Mokhtar N. Treatment effects of two different appliances on pharyngeal airway space in mixed dentition Malay children. Int J Pediatr Otorhinolaryngol. 2019;125:159-63. | Excluded; no untreated Class III group |
| 516 | Graber LW. Hyoid changes following orthopedic treatment of mandibular prognathism. Angle Orthod. 1978;48(1):33-8. | Excluded; no untreated Class III group |
| 517 | Hiyama S, Suda N, Ishii-Suzuki M, Tsuiki S, Ogawa M, Suzuki S, et al. Effects of Maxillary Protraction on Craniofacial Structures and Upper-Airway Dimension. Angle Orthod. 2002;72(1):43-7. | Excluded; no untreated Class III group |

| | | |
|---|---|---|
| 518 | Hwang DM, Lee JY, Choi YJ, Hwang CJ. Evaluations of the tongue and hyoid bone positions and pharyngeal airway dimensions after maxillary protraction treatment. Cranio. 2018:1-9. | Excluded; no untreated Class III group |
| 519 | Hwang DM, Lee JY, Choi YJ, Hwang CJ. Evaluations of the tongue and hyoid bone positions and pharyngeal airway dimensions after maxillary protraction treatment. Cranio. 2019;37(4):214-22. | Excluded; no untreated Class III group |
| 520 | Katyal V, Wilmes B, Nienkemper M, Darendeliler MA, Sampson W, Drescher D. The efficacy of Hybrid Hyrax-Mentoplate combination in early Class III treatment: a novel approach and pilot study. Australian Orthodontic Journal. 2016;32(1):88-96. | Excluded; no untreated Class III group |
| 521 | Kaygisiz E, Tuncer BB, Yuksel S, Tuncer C, Yildiz C. Effects of maxillary protraction and fixed appliance therapy on the pharyngeal airway. Angle Orthod. 2009;79(4):660-7. | Excluded; no untreated Class III group |
| 522 | Lee JW, Park KH, Kim SH, Park YG, Kim SJ. Correlation between skeletal changes by maxillary protraction and upper airway dimensions. Angle Orthod. 2011;81(3):426-32. | Excluded; no untreated Class III group |
| 523 | Lee KM, Chung DH, Lee JW, Lee SM. Cervical vertebrae maturation, dentoalveolar, head postural and respiratory parameters in predicting the stable outcome of face-mask treatment. Eur J Orthod. 2015;37(3):251-6. | Excluded; no untreated Class III group |
| 524 | Mitani Y, Banabilh SM, Singh GD. Craniofacial changes in patients with Class III malocclusion treated with the RAMPA system. Int J Orthod Milwaukee. 2010;21(2):19-25. | Excluded; no untreated Class III group |
| 525 | Nguyen T, De Clerck H, Wilson M, Golden B. Effect of Class III bone anchor treatment on airway. Angle Orthod. 2015;85(4):591-6. | Excluded; no untreated Class III group |
| 526 | Oktay H, Ulukaya E. Maxillary protraction appliance effect on the size of the upper airway passage. Angle Orthod. 2008;78(2):209-14. | Excluded; no untreated Class III group |
| 527 | Onem Ozbilen E, Yilmaz HN, Kucukkeles N. Comparison of the effects of rapid maxillary expansion and alternate rapid maxillary expansion and constriction protocols followed by facemask therapy. Korean J Orthod. 2019;49(1):49-58. | Excluded; no untreated Class III group |
| 528 | Pamporakis P, Nevzatoglu S, Kucukkeles N. Three-dimensional alterations in pharyngeal airway and maxillary sinus volumes in Class III maxillary deficiency subjects undergoing orthopedic facemask treatment. Angle Orthod. 2014;84(4):701-7. | Excluded; no untreated Class III group |
| 529 | Sayinsu K, Isik F, Arun T. Sagittal airway dimensions following maxillary protraction: a pilot study. Eur J Orthod. 2006;28(2):184-9. | Excluded; no untreated Class III group |
| 530 | Akin M, Ucar FI, Chousein C, Sari Z. Effects of chincup or facemask therapies on the orofacial airway and hyoid position in Class III subjects. J Orofac Orthop. 2015;76(6):520-30. | Included |
| 531 | Baccetti T, Franchi L, Mucedero M, Cozza P. Treatment and post-treatment effects of facemask therapy on the sagittal pharyngeal dimensions in Class III subjects. Eur J Orthod. 2010;32(3):346-50. | Included |
| 532 | Balos Tuncer B, Ulusoy C, Tuncer C, Turkoz C, Kale Varlik S. Effects of reverse headgear on pharyngeal airway in patients with different vertical craniofacial features. Braz Oral Res. 2015;29. | Included |
| 533 | Cretella Lombardo E, Franchi L, Lione R, Chiavaroli A, Cozza P, Pavoni C. Evaluation of sagittal airway dimensions after face mask therapy with rapid maxillary expansion in Class III growing patients. Int J Pediatr Otorhinolaryngol. 2020;130:109794. | Included |
| 534 | Danaei SM, Ajami S, Etemadi H, Azadeh N. Assessment of the effect of maxillary protraction appliance on pharyngeal airway dimensions in relation to changes in tongue posture. Dent Res J 2018;15:208-14. | Included |
| 535 | Kilinc AS, Arslan SG, Kama JD, Ozer T, Dari O. Effects on the sagittal pharyngeal dimensions of protraction and rapid palatal expansion in Class III malocclusion subjects. Eur J Orthod. 2008;30(1):61-6. | Included |
| 536 | Menendez-Diaz I, Muriel J, Cobo JL, Alvarez C, Cobo T. Early treatment of Class III malocclusion with facemask therapy. Clin Exp Dent Res. 2018;4(6):279-83. | Included |
| 537 | Mucedero M, Baccetti T, Franchi L, Cozza P. Effects of maxillary protraction with or without expansion on the sagittal pharyngeal dimensions in Class III subjects. Am J Orthod Dentofacial Orthop. 2009;135(6):777-81. | Included |
| 538 | Tuncer BB, Kaygisiz E, Tuncer C, Yuksel S. Pharyngeal airway dimensions after chin cup treatment in Class III malocclusion subjects. J Oral Rehabil. 2009;36(2):110-7. | Included |
| 539 | Yagci A, Uysal T, Usumez S, Orhan M. Effects of modified and conventional facemask therapies with expansion on dynamic measurement of natural head position in Class III patients. Am J Orthod Dentofacial Orthop. 2011;140(5):e223-31. | Included |

**Supplementary Material S5**. Results of individual studies comparing maxillary protraction to untreated controls that are not included in meta-analyses.

| Nr | Study | Outcome* | MD (95% CI) | P | CR |
|---|---|---|---|---|---|
| 1 | Kilinc 2008 | Total airway area ($mm^2$) | 222.86 (14.04, 431.68) | **0.04** | **Yes** |
| 2 | Tuncer 2015 | Nasopharynx: AA'-Pm' (mm) | 1.55 (0.65, 2.46) | **0.001** | **No** |
| 3 | Tuncer 2015 | Nasopharynx: Pm'-SPL (mm) | -1.25 (-2.37, -0.14) | **0.03** | **No** |
| 4 | Tuncer 2015 | Nasopharynx: S-PNS (mm) | 0.15 (-0.95, 1.25) | 0.79 | - |
| 5 | Mucedero 2009; Baccetti 2010 | Nasopharynx: PNS-Ba (mm) | 1.72 (0.24, 3.20) | **0.02** | **No** |
| 6 | Mucedero 2009; Baccetti 2010 | Nasopharynx: PNS-H (mm) | 1.41 (0.24, 2.59) | **0.02** | **No** |
| 7 | Mucedero 2009; Baccetti 2010 | Nasopharynx: Ptm-Ba (mm) | 1.34 (0.26, 2.42) | **0.02** | **No** |
| 8 | Tuncer 2015 | Oropharynx: eb-Peb (mm) | 1.25 (0.09, 2.41) | **0.04** | **No** |
| 9 | Tuncer 2015 | Oropharynx: IPS (mm) | 0.05 (-1.16, 1.26) | 0.94 | - |
| 10 | Tuncer 2015 | Oropharynx: MPS (mm) | 1.20 (0.39, 2.01) | **0.004** | **No** |
| 11 | Tuncer 2015 | Oropharynx: AA-PNS (mm) | 0.40 (-0.51, 1.31) | 0.39 | - |
| 12 | Tuncer 2015 | Oropharynx: SPS (mm) | 0.35 (-0.56, 1.26) | 0.45 | - |
| 13 | Tuncer 2015 | Oropharynx: ve-Pve (mm) | 1.05 (0.19, 1.91) | **0.02** | **No** |
| 14 | Kilinc 2008 | Oropharynx: APW-PPW (mm) | 1.27 (-0.83, 3.37) | 0.24 | - |
| 15 | Kilinc 2008 | Oropharynx: APW'-PPW' (mm) | 5.00 (0.75, 9.25) | **0.02** | **Yes** |

* from explanation of each landmark, consult the original studies.

CI, confidence interval; CR, clinically relevant (judged as effect being larger than one standard deviation of the control group pre-treatment); MD, mean difference.

**Supplementary Material S6**. Contour-enhanced forest plot for the comparison of maxillary protraction with facemask versus changes in untreated controls in terms of total nasopharyngeal area.

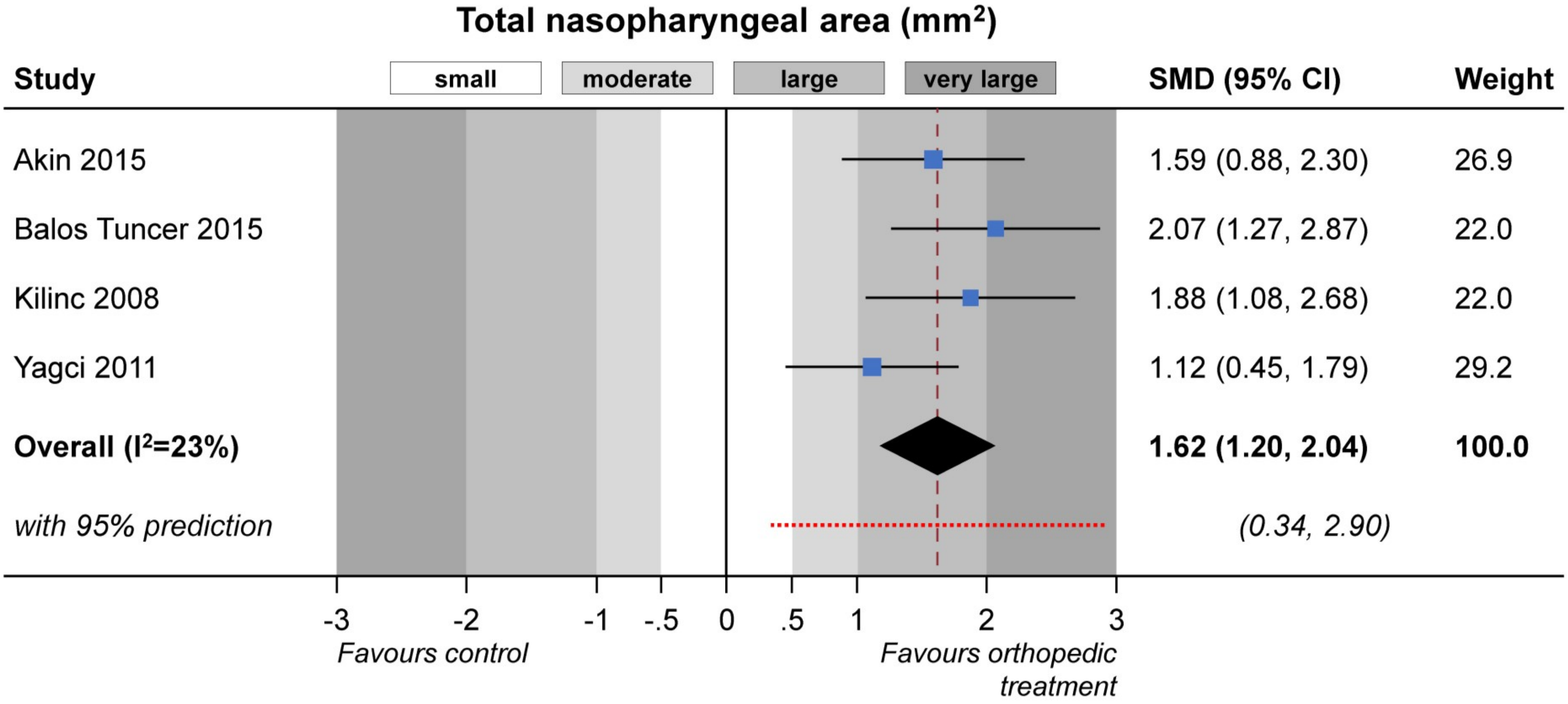

**Supplementary Material S7**. Contour-enhanced forest plot for the comparison of maxillary protraction with facemask versus changes in untreated controls in terms of upper airway dimension.

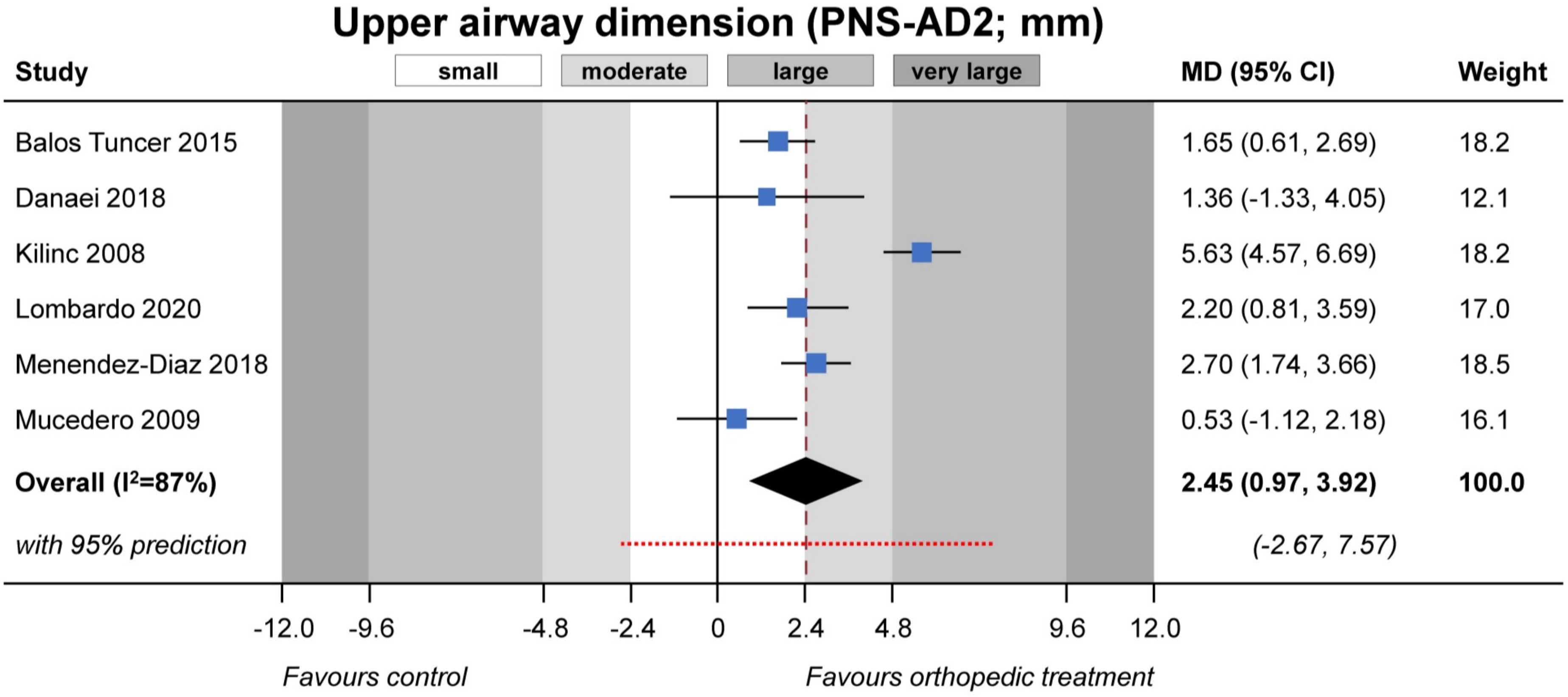

**Supplementary Material S8**. Contour-enhanced forest plot for the comparison of maxillary protraction with facemask versus changes in untreated controls in terms of lower airway dimension.

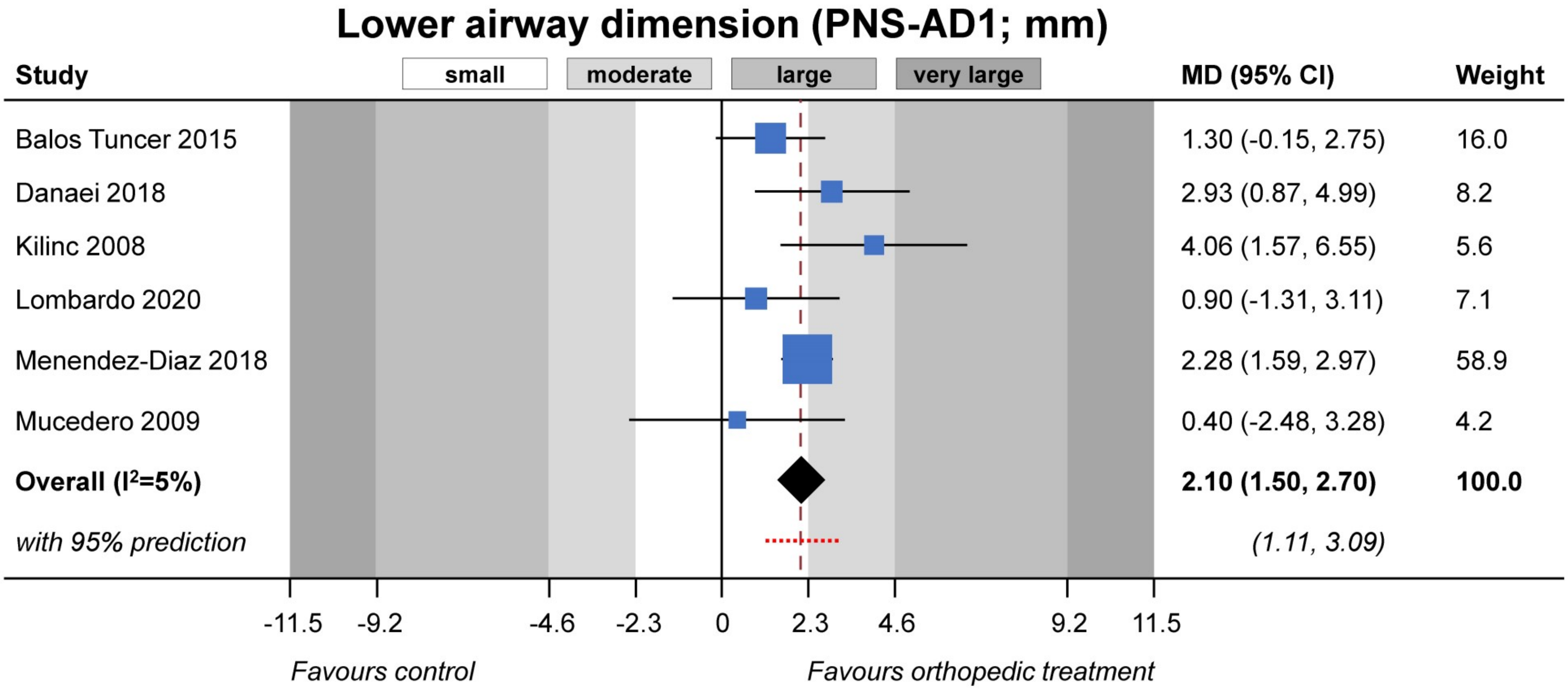

**Supplementary Material S9**. Contour-enhanced forest plot for the comparison of maxillary protraction with facemask versus changes in untreated controls in terms of McNamara's upper pharynx dimension.

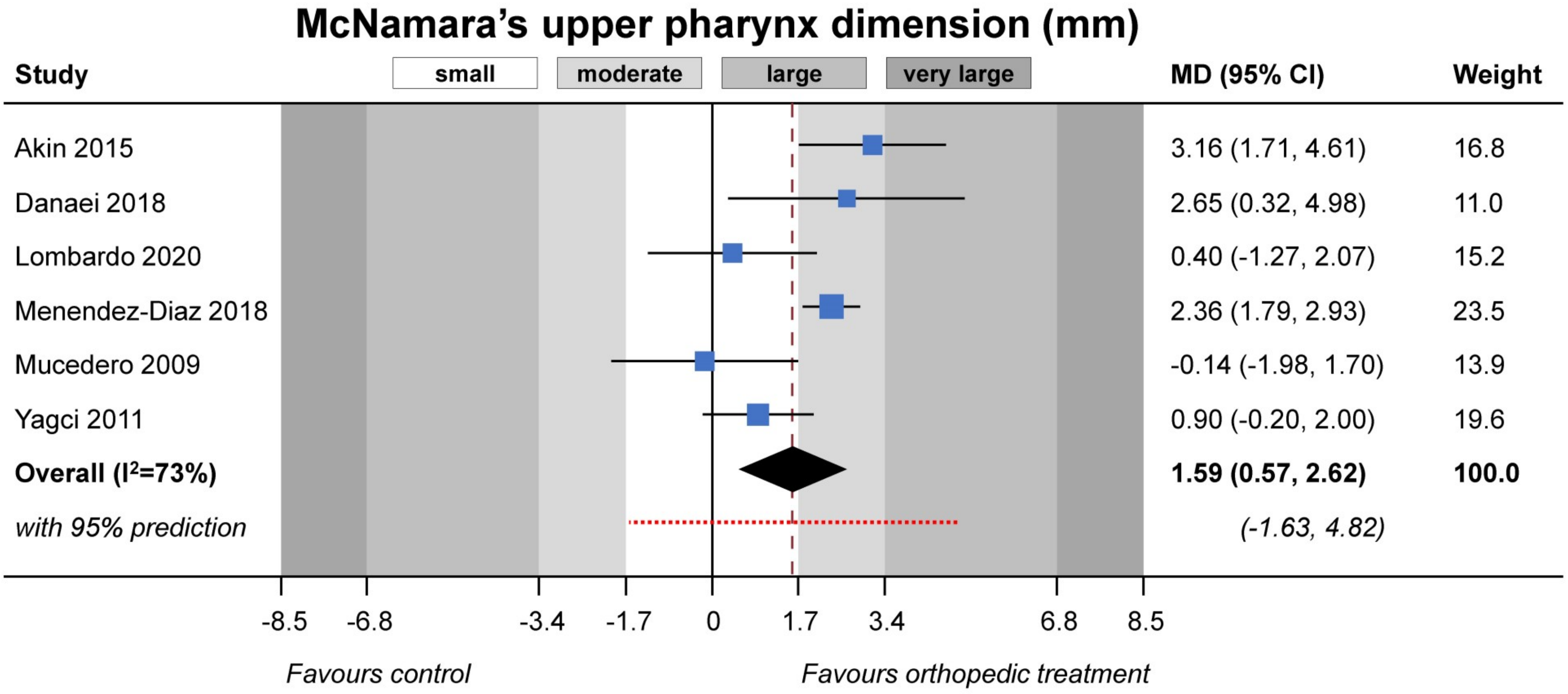

**Supplementary Material S10**. Contour-enhanced forest plot for the comparison of maxillary protraction with facemask versus changes in untreated controls in terms of McNamara's lower pharynx dimension.

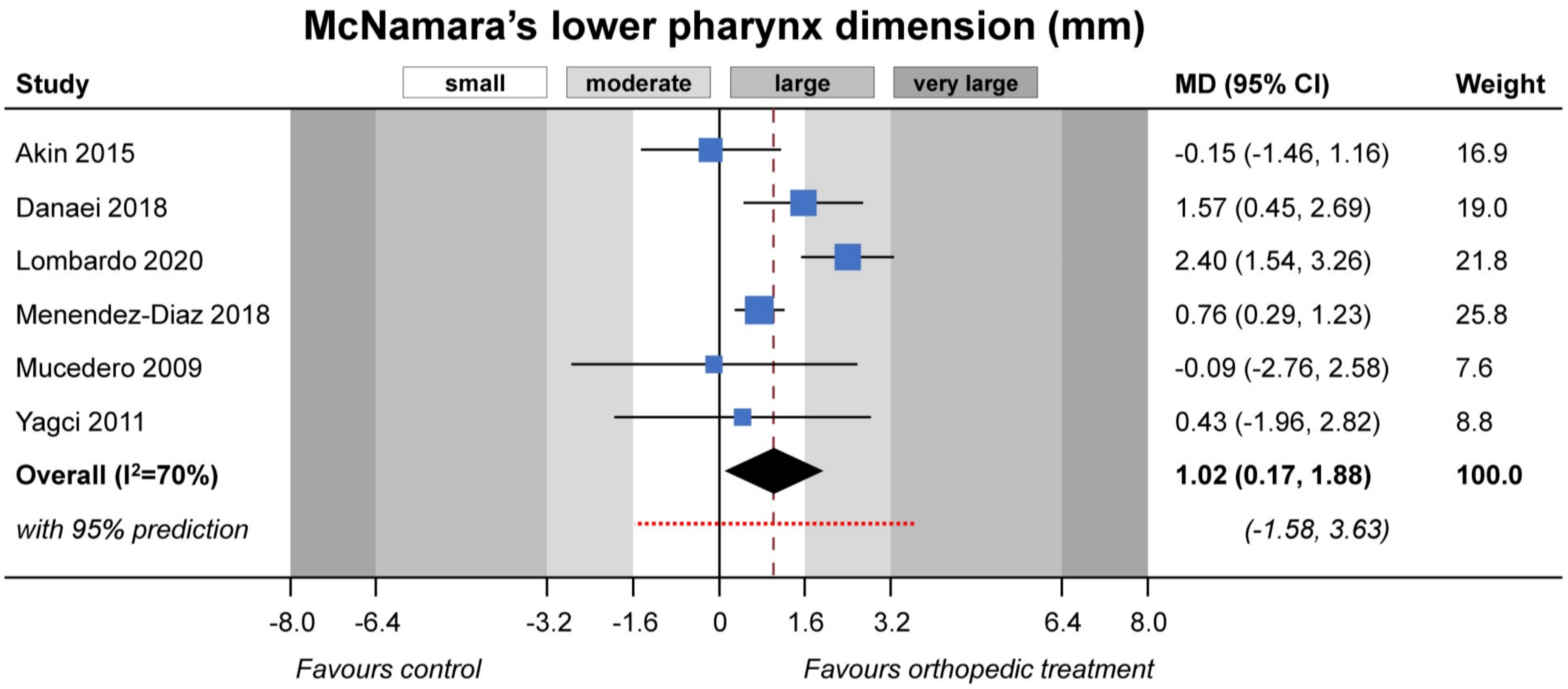

**Supplementary Material S11**. Results of individual studies comparing chincup to untreated controls that are not included in meta-analyses.

| Nr | Study | Outcome* | MD (95% CI) | P | CR |
|---|---|---|---|---|---|
| 1 | Akin 2015 | Nasopharyngeal area (adenoidal) ($mm^2$) | 12.29 (-16.22, 40.80) | 0.40 | - |
| 2 | Akin 2015 | Nasopharyngeal area (aerial) ($mm^2$) | -3.22 (-44.23, 37.79) | 0.88 | - |
| 3 | Akin 2015 | Nasopharyngeal area (total) ($mm^2$) | 9.08 (-23.74, 41.90) | 0.59 | - |
| 4 | Tuncer 2009 | Nasopharyngeal area ($mm^2$) | 10183.00 (10074.25, 10291.75) | **<0.001** | Yes |
| 5 | Tuncer 2009 | Nasopharynx: AA'-Pm' (mm) | 0.75 (-0.38, 1.88) | 0.19 | - |
| 6 | Tuncer 2009 | Nasopharynx: Pm'-SPL (mm) | -0.28 (-1.97, 1.41) | 0.75 | - |
| 7 | Tuncer 2009 | Nasopharynx: PNS-AD1 (mm) | 1.59 (-0.95, 4.13) | 0.22 | - |
| 8 | Tuncer 2009 | Nasopharynx: PNS-AD2 (mm) | -0.20 (-2.42, 2.02) | 0.86 | - |
| 9 | Tuncer 2009 | Nasopharynx: PNS-S (mm) | 0.35 (-1.01, 1.71) | 0.61 | - |
| 10 | Tuncer 2009 | Oropharyngeal area ($mm^2$) | -8231.00 (-10616.52, -5845.48) | **<0.001** | No |
| 11 | Tuncer 2009 | Oropharynx: AA-PNS (mm) | -0.18 (-1.94, 1.58) | 0.84 | - |
| 12 | Tuncer 2009 | Oropharynx: eb-Peb (mm) | 1.62 (-1.23, 4.47) | 0.27 | - |
| 13 | Tuncer 2009 | Oropharynx: IPS (mm) | 1.66 (-0.67, 3.99) | 0.16 | - |
| 14 | Tuncer 2009 | Oropharynx: MPS (mm) | 0.79 (-0.76, 2.34) | 0.32 | - |
| 15 | Tuncer 2009 | Oropharynx: SPS (mm) | 1.13 (-0.48, 2.74) | 0.17 | - |
| 16 | Tuncer 2009 | Oropharynx: ve-Pve (mm) | 0.57 (-0.75, 1.89) | 0.40 | - |
| 17 | Akin 2015 | Lower pharynx dimension (mm) | -0.40 (-1.68, 0.88) | 0.54 | - |
| 18 | Akin 2015 | Upper pharynx dimension (mm) | -0.25 (-1.61, 1.11) | 0.72 | - |

* from explanation of each landmark, consult the original studies.

CI, confidence interval; CR, clinically relevant (judged as effect being larger than one standard deviation of the control group pre-treatment); MD, mean difference.

**Supplementary Material S12**. Sensitivity analyses of meta-analyses post-treatment with at least 3 studies.

| | | Sample size | | | Baseline similarity (airways) | | |
|---|---|---|---|---|---|---|---|
| | Original | Large | Small | | Similar | Different | |
| **Outcome** | **Effect (95% CI)** | **Effect (95% CI)** | **Effect (95% CI)** | **P** | **Effect (95% CI)** | **Effect (95% CI)** | **P** |
| Total nasopharyngeal area – ($mm^2$) | n=4 SMD=1.62 (1.20, 2.04) | n=0 | n=4 | - | n=2 SMD=1.97 (1.41, 2.54) | n=2 MD=1.34 (0.86, 1.83) | 0.14 |
| Upper adenoid size (AD2-H; mm) | n=3 MD=0.59 (-0.52, 1.70) | n=3 | n=0 | - | n=3 | n=0 | - |
| Lower adenoid size (AD1-Ba; mm) | n=3 MD=0.12 (-2.20, 2.44) | n=3 | n=0 | - | n=3 | n=0 | - |
| Upper airway dimension (PNS-AD2; mm) | n=6 MD=2.45 (0.97, 3.92) | n=3 MD=1.95 (0.74, 3.16) | n=3 MD=3.00 (0.21, 5.79) | 0.45 | n=6 | n=0 | - |
| Lower airway dimension (PNS-AD1; mm) | n=6 MD=2.10 (1.50, 2.70) | n=3 MD=1.71 (0.50, 2.92) | n=3 MD=2.52 (0.90, 4.14) | 0.84 | n=6 | n=0 | - |
| McNamara's upper pharynx dimension (mm) | n=6 MD=1.59 (0.57, 2.62) | n=3 MD=1.07 (-0.55, 2.69) | n=3 MD=2.11 (0.58, 3.65) | 0.90 | n=4 MD=1.38 (0.04, 2.73) | n=2 MD=1.98 (-0.23, 4.19) | 0.80 |
| McNamara's lower pharynx dimension (mm) | n=6 MD=1.02 (0.17, 1.88) | n=3 MD=1.26 (-0.10, 1.88) | n=3 MD=0.70 (-0.52, 1.93) | 0.75 | n=4 MD=1.37 (0.43, 2.30) | n=2 MD=-0.02 (-1.17, 1.14) | 0.34 |

CI, confidence interval; MD, mean difference; SMD, standardized mean difference.
